# Parenteral clomipramine for depression or obsessive-compulsive disorder: a systematic review and meta-analysis

**DOI:** 10.1017/neu.2026.10074

**Published:** 2026-04-14

**Authors:** Michael Ioannou, Örjan Falk, Joss Gustavsson, Johan Nilsson, Petteri Sjögren, Steinn Steingrimsson, Therese Svanberg, Zoltán Szabó, Susanna M. Wallerstedt

**Affiliations:** 1 Institute of Neuroscience and Physiology, University of Gothenburg Sahlgrenska Academy, Sweden; 2 Psykiatri Affektiva, Sahlgrenska University Hospital, Sweden; 3 Medical Library, Sahlgrenska University Hospital, Sweden; 4 Department of Clinical Pharmacology, Sahlgrenska University Hospital, Sweden; 5 Center for Health Technology Assessment, Sahlgrenska University Hospital, Sweden; 6 Department of Pharmacology, University of Gothenburg Sahlgrenska Academy, Sweden

**Keywords:** clomipramine, depression, efficacy, obsessive-compulsive disorder, parenteral

## Abstract

In severe cases of depression and obsessive-compulsive disorder (OCD), clomipramine is sometimes administered parenterally. This systematic review aimed to investigate whether parenteral clomipramine is superior to oral clomipramine or other treatments, primarily in terms of reducing depressive/OCD symptoms within two weeks (CRD420250654029). Medline, Embase, the Cochrane Library, and PsycInfo were searched for relevant publications. Randomized controlled trials (RCTs) without a high risk of bias formed the primary basis for the conclusions. Meta-analyses were performed when applicable. Certainty of evidence was assessed according to GRADE. The literature search identified 4973 unique publications, whereof 14 RCTs contributed data regarding the question at issue in this systematic review. The evidence synthesis revealed that parenteral clomipramine may not be superior to oral administration in terms of reducing depressive symptoms within two weeks, but a clinically relevant effect cannot be excluded (low certainty of evidence; five RCTs including 70 patients; mean difference of change in Hamilton depression rating scale scores (meta-analysis based on three RCTs): −1.27 (95% confidence interval: −3.09 to 0.54; 2, I2 = 22%). Regarding patients with OCD, no conclusion could be drawn (very low certainty of evidence; two RCTs including 47 patients; meta-analysis not conducted due to heterogeneity). Regarding comparisons with other treatments, the available RCT (depression) did not allow for conclusions, or no RCTs (OCD) were available. Current evidence indicates that parenteral administration of clomipramine may not be favourable compared to oral administration, and RCTs with relevant comparisons such as electroconvulsive therapy and ketamine are lacking.

Summations

- In depression, parenteral clomipramine does not show clear short- or long-term advantages over oral clomipramine in symptom reduction or treatment discontinuation, although a short-term benefit cannot be ruled out due to imprecision of effect estimates.
- In obsessive-compulsive disorder(OCD), current randomised trials provide very uncertain evidence, and no superiority of parenteral clomipramine over oral clomipramine or placebo has been demonstrated.

Considerations

- The certainty of the evidence is predominantly low or very low, mainly due to small sample sizes, few events, and wide confidence intervals(CIs) in the included randomised controlled trials(RCTs).
- Important clinically relevant outcomes such as suicidality, functioning, hospital length of stay, and quality of life are lacking.

## Introduction

The tricyclic antidepressant (TCA) clomipramine has been administered parenterally for decades because of hypothesised advantages over oral formulations and other treatments. Arguments for parenteral over oral administration include more predictable bioavailability; a higher parent-to-metabolite ratio with serotonergic activity being more prominent in the parent drug; faster achievement of steady state, potentially leading to a quicker onset of action; fewer compliance issues; and a potential placebo effect related to the mode of administration (Evans *et al*., 1980; Balant-Gorgia *et al*., 1991; Hendset *et al*., 2006; Jones *et al*., 2021; Peciña *et al*., 2018). This may be particularly relevant in inpatient care, where rapid symptom relief and reduction of suicide risk are critical treatment goals (Zisler *et al*., 2024; Abdolizadeh *et al*., 2025). Furthermore, it has been suggested that TCAs may be more effective than selective serotonin reuptake inhibitors (SSRIs) in treating depression and obsessive–compulsive disorder (OCD) (Anderson, 2000; Cohen *et al*., 2024b).

Guidelines acknowledge the potential of parenteral clomipramine (Katzman *et al*., 2014). However, its global use remains unclear. A recent nationwide survey showed that 31 of 37 responding Swedish clinics had used parenteral clomipramine during the past three years, and 29 clinics (78%) reported considering it an alternative to electroconvulsive therapy (ECT) in certain cases (Ioannou *et al*., 2025). Patients receiving this treatment, therefore, likely represent particularly challenging cases for clinical decision-making. In Sahlgrenska University Hospital, serving 1.8 million inhabitants in the second largest region in Sweden, 4.1% of adult patients admitted for at least six days with a depressive state between 2016 and 2022 received such treatment (Ioannou *et al*., 2025).

Despite decades of clinical use, critical evidence gaps regarding the clinical effects of parenteral clomipramine persist. To our knowledge, compilations of evidence regarding the efficacy in depression and OCD are limited to older publications that lack meta-analyses, do not assess the risk of bias in the included studies, and do not evaluate the certainty of the evidence (Moukaddam & Hirschfeld, 2004; Ravindran *et al*., 2010). This systematic review aimed to fill these gaps by examining whether parenteral clomipramine is more effective than oral clomipramine, other treatments, or placebo in reducing depression or OCD symptoms and other outcomes in patients with these conditions.

## Methods

The current systematic review was conducted as part of a health technology assessment (HTA), intended to provide information for regional evidence-based decision-making and also including, for instance, the number of patients at issue in the region as well as economic aspects (Ioannou *et al*., 2025). It is reported according to the Preferred Reporting Items for Systematic Reviews and Meta-Analyses (PRISMA) guidelines (Page *et al*., 2021) and was registered with PROSPERO (CRD420250654029).

The research question was formulated using the PICO framework. Accordingly, participants (P) were patients with depression (P1) or OCD (P2). Predefined subgroups of P were (i) older patients (≥65 years) and (ii) severe depression with melancholic or psychotic symptoms. The intervention (I) was parenteral clomipramine. Predefined subgroups of I were monotherapy and add-on therapy. The comparison (C) was oral clomipramine (C1), other treatment (C2), or placebo (C3). Predefined subgroups of C2 were ECT and ketamine. Although a placebo is not a clinically relevant treatment alternative, we included this comparison to capture the entire evidence base for parenteral clomipramine. The main outcomes (O) were depressive symptoms (P1) or OCD symptoms (P2) within two weeks. Additional outcomes were long-term (>2 weeks) depressive symptoms (P1) or OCD symptoms (P2), suicide attempt, all-cause mortality, suicidal ideation, global functioning, treatment discontinuation, length of hospital stay, health-related quality of life (HRQL), and adverse events related to administration via injection/infusion (e.g., thrombophlebitis, infection, or anaphylaxis). Publications were restricted to RCTs, and languages were limited to English, Swedish, Danish, and Norwegian.

### Literature search and study selection

On 20 September 2024, two experienced medical librarians (J.G. and T.S.) conducted systematic searches in Medline, Embase, the Cochrane Library, and PsycInfo. Search strategies are provided in the supplement. Websites of Scandinavian national and regional HTA organisations were visited in April 2025. A citation search – both backward and forward – of all included articles, as well as a selection of excluded systematic reviews, was conducted in Web of Science Core Collection.

The titles and abstracts retrieved in the systematic literature search were independently screened by two authors (J.G. and T.S.) regarding eligibility for full-text retrieval, i.e., those not clearly outside the scope of this systematic review. This screening process was conducted using the Rayyan tool (Ouzzani *et al*., 2016). Any disagreements were resolved through consensus discussions. All retrieved full-text reports were independently read by at least two authors (J.G. and T.S., or M.I., Ö.F., J.N., P.S., S.S., and S.M.W.). In a consensus meeting, we made the final decision on which reports to include in this systematic review, i.e., those that fulfilled the PICO criteria. For articles excluded in consensus, after full-text reading, reasons for exclusion were noted.

A search on Clinicaltrials.gov (March 4, 2025), using the search terms (chlorimipramine OR chlomipramine OR anafranil OR hydiphen OR clomipramine), identified 37 trials, none of which met the PICO criteria.

### Data extraction and study assessments

Data from the included studies were independently extracted by three authors (S.M.W., M.I., and J.N.), and any discrepancies were resolved through consensus discussions. Data collection included study design, participant details, characteristics of individuals in the intervention and control groups, treatment regimen information, and results related to the outcomes of interest.

For the outcome regarding depressive and OCD symptoms within two weeks, we chose to extract the results closest to one week and two weeks, pooling the latter if data were available; otherwise, we used the former. For the outcome regarding depressive and OCD symptoms after more than two weeks, we chose to extract the results that were closest to four weeks and pooled them if possible. For P1, the change in the Hamilton Depression Rating Scale (HDRS) was beforehand determined to be the primary basis for conclusions. Similarly, for P2, the change in the Yale-Brown Obsessive Compulsive Scale (Y-BOCS) was the primary basis for conclusions. When mean scores were not provided, we calculated them from individual data if available. When exact scores were not available in the text, we estimated them from figures if available. When the standard deviation (SD) for the change was not provided, we estimated this figure using the following formula: sqrt(((n-1)×SDpre2 + (n-1)×SDpost2)/(2n/2)). When the change in summarised HDRS scores was not available, we extracted separate HDRS scores or Montgomery Åsberg Depression Rating Scale (MADRS) scores. We did not request additional data from the study investigators.

Each study was independently assessed by at least five authors regarding the risk of bias and directness (M.I., J.N., P.S., S.M.W., and either Ö.F., S.S., or Z.S.), followed by consensus discussions. We used the Cochrane risk of bias tool for randomised trials (RoB 2) (Sterne *et al*., 2019). Beforehand, we decided not to judge RCTs too harshly on reporting aspects that were not applicable at the time of conduct, such as reporting of random sequence generation. Directness was assessed based on a checklist developed regionally, including questions about each component of the PICO, and the extent to which the assessed study corresponded to this (Center for Health Technology Assessment 2021). The reasons behind the consensus assessments of risk of bias and directness were recorded.

The certainty of evidence was assessed using the Grading of Recommendations Assessment, Development and Evaluation (GRADE) approach (Atkins *et al*., 2004). Beforehand, we decided that studies without a high risk of bias would constitute the primary basis for conclusions. In assessments regarding precision, we took into account whether the 95% confidence interval (CI) included the minimum clinically important difference (MCID) of 2 for HDRS scores (Hengartner & Plöderl, 2022) and 4.9 for Y-BOCS scores (Cohen *et al*., 2024a). Informative statements according to GRADE guidelines were used to summarise the results (Santesso *et al*., 2020).

### Meta-analysis

When two or more studies provided data that could be pooled, random effects meta-analyses were performed to obtain weighted mean differences, including 95% CI, using the Review Manager (RevMan) version 5.4.1 software (Nordic Cochrane Centre, Cochrane Collaboration, Copenhagen, Denmark). We pooled HDRS data if a summarised result, including the items at issue, was reported, irrespective of abbreviations and item selections that were used, representing the development of this scale over the years. For dichotomous outcomes, the Peto odds ratio was used in case of zero-event arms, as this method does not require corrections (Higgins *et al*., 2024). Heterogeneity was assessed with I2 statistics.

## Results

**Figure 1.**
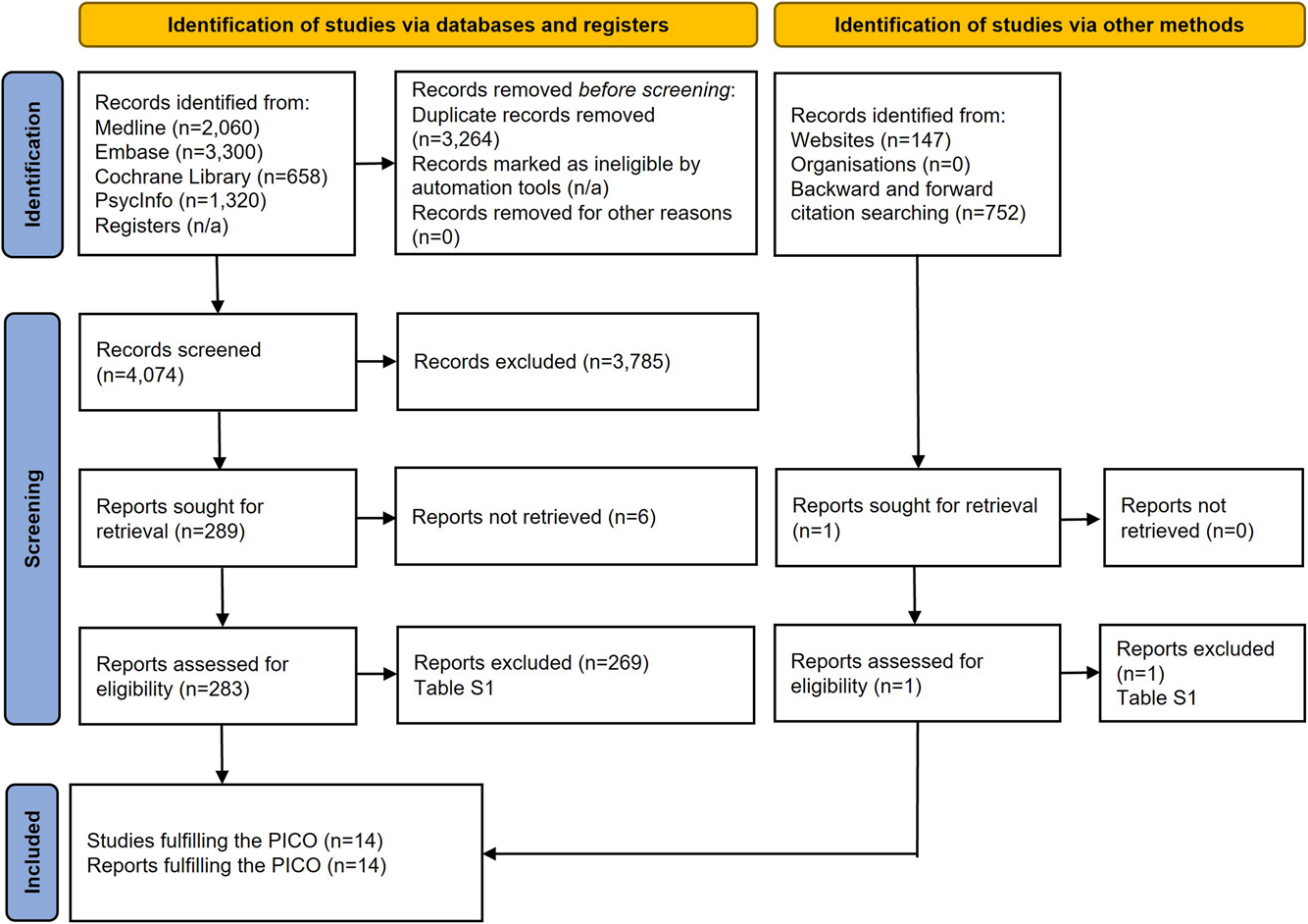
PRISMA flowchart.

After removal of duplicates, the literature search identified 4,973 unique publications, 14 of which met the PICO criteria of this systematic review (Figure 1)

(Escobar *et al*., 1973; Hordern *et al*., 1979; Lecrubier *et al*., 1980; de Cuyper *et al*., 1981; Drago *et al*., 1983; Fähndrich, 1983; Faravelli *et al*., 1983; Pollock *et al*., 1989; Spreux-Varoquaux *et al*., 1996; Koran *et al*., 1997, 2006; Sallee *et al*., 1997; Fallon *et al*., 1998; Altamura *et al*., 2008). Publications excluded after full-text reading, as well as the reasons for excluding them, are presented in Table S1.

### Study characteristics

The included RCTs, published between 1973 and 2008, are summarised in Table 1. They were performed in the United States (Escobar *et al*., 1973; Pollock *et al*., 1989; Koran *et al*., 1997, 2006; Sallee *et al*., 1997; Fallon *et al*., 1998), Italy (Drago *et al*., 1983; Faravelli *et al*., 1983; Altamura *et al*., 2008), France (Lecrubier *et al*., 1980; Spreux-Varoquaux *et al*., 1996), Belgium (de Cuyper *et al*., 1981), Germany (Fähndrich, 1983),and the United Kingdom (Hordern *et al*., 1979). Ten RCTs were double-blind (Escobar *et al*., 1973; Hordern *et al*., 1979; Drago *et al*., 1983; Faravelli *et al*., 1983; Pollock *et al*., 1989; Spreux-Varoquaux *et al*., 1996; Koran *et al*., 1997, 2006; Sallee *et al*., 1997; Fallon *et al*., 1998), two were single-blind (Lecrubier *et al*., 1980; Altamura *et al*., 2008), and two were open-label (de Cuyper *et al*., 1981; Fähndrich, 1983).

**Table 1.** Summary of study characteristics and findings of the included studies

Authors country	Design	Patients	Treatment regimen, I and C	Findings: I vs. C
Altamura *et al*., 2008 Italy	Single-blind RCT, blinded raters	MDD/BPD with MDE, outpatients, cut-off symptom scores not defined. I: *n* = 18; C2: *n* = 18; C3: *n* = 18. Sex in randomisation groups NR, in total 16 women. Age in randomisation groups NR.	I: Clomipramine IV (25 mg/day) C2: Citalopram IV (10 mg/day) or C3: saline. I and C2: Treatment duration: 5 days Add-on therapy: maintained on treatment with an SSRI.	Depressive symptoms within 2 weeks; mean change in HAM-D_21_ scores: I: −11_day 5_ (initial score: 20) vs. C2: −12_day 5_ (initial score: 24) and C3: −2.5_day 5_ (initial score: 20). No treatment discontinuations.
De Cuyper *et al*., 1981 Belgium	Open label RCT	Outpatients. recently hospitalised for vital depressive syndrome, cut-off symptom scores not defined. I: 5 (3 women, age range: 23–43 years); C1: 5 (3 women, age range: 35–50 years).	I: Clomipramine IM (gradual titration; 25 mgx2 initially, after 2 days 50 mgx2, after 14 days, switch to oral daily dose of 200 mg administered in three separate doses) C1: Clomipramine PO (25 mgx3, with gradual increase to 200 mg daily on day 6). I and C1: Treatment duration: 4 weeks Monotherapy (no other drugs allowed, apart from benzodiazepines as hypnotic).	Depressive symptoms within 2 weeks; mean (SD) change in HRS scores _week 2_: I: −7.2 (4.6) vs. C1: −9 (3.5). Long-term depressive symptoms; mean (SD) change in HRS scores_week 4_: I: I: −10.2 (7.0) vs. C1: −15 (9.7). (Absolute HRS scores NR)
Drago *et al*., 1983 Italy	Double-blind RCT	Hospitalised with severe primary depression (HDRS ≥18 or overt suicide tendency), treatment resistant (to 3 antidepressants over the previous 6 months. I: *n* = 20 (all women, mean (SD) age: 49.35 (10.43) years) C2: *n* = 20 (all women, mean (SD) age: 48.45 (13.12)).	I: Clomipramine IV (100 mg daily) C2: maprotiline IV (100 mg daily). Treatment duration: 21 days (9 days if non-responder, thereafter cross-over). Add-on or monotherapy NR.	Depressive symptoms within 2 weeks; mean (SD) change in HDRS_17_ scores: I: −7.25 (8.08)_day 5_, −12.25 (10.59)_day 9_ (initial score: 25.4) vs. C2: −5.1 (5.92)_day 5_, −7.8 (9.83)_day 9_ (initial score: 26.6) (Long-term depressive symptoms reported as above, but non-responders crossed over after 9 days and the results are therefore not relevant.)
Escobar *et al*., 1973 United States	Double-blind RCT, double dummy	Patients with ‘target symptom of depression’, HDS: ≥25. I: *n* = 14 (9 women); C1: *n* = 17 (9 women); after dropouts (NR) were replaced. Age in randomisation groups NR, overall mean (range): 43 (20–64) years.	I: Clomipramine IV (day 1–10: gradual titration, not explicitly described, 25–150 mg) C1: Clomipramine PO (day 1–10: gradual titration not explicitly described, 75–300 mg). Both I and C, day 11–21: Clomipramine PO, 75–300 mg. Add-on or monotherapy NR.	Depressive symptoms within 2 weeks; mean HDS scores (not summarised): I: sleep disturbance, 1.4_day 7_, 1.3_day 10_; somatisation, 1.9_day 7_, 1.6_day 10_; anxiety-depression, 1.8_day 7_, 1.7_day 10_; apathy, 1.8_day 7_, 1.7_day 10_ vs. C1: sleep disturbance, 1.5_day 7_, 1.5_day 1_; somatisation, 1.5_day 7_, 1.5_day 10_; anxiety-depression, 1.8_day 7_, 1.6_day 10_; apathy, 2.0_day 7_, 2.0_day 10_ Long-term depressive symptoms; mean HRS scores_day 28_ (not summarised): I: sleep disturbance, 1.4; somatisation, 1.7; anxiety-depression, 1.6; apathy, 1.6 vs. C1: sleep disturbance, 1.5: somatisation, 1.7; anxiety-depression, 1.8; apathy, 1.9. Adverse events related to administration via injection/infusion: I: 2/14 (treatment withheld due to orthostatic hypotension) vs. C1: 0/17.
Fallon *et al*., 1998 United States	Double-blind RCT	OCD poorly responsive to clomipramine PO, Y-BOCS>16. I: *n* = 29; C3: *n* = 25. Sex and age in randomisation groups NR, in total 33 women; mean (SD) age: 32.4 (9) years.	I: Clomipramine IV (gradual increments: 25 mg/day for 8 days, thereafter 250 mg) C3: Placebo. I and C3: Treatment duration: 14 weekdays. Monotherapy (2-week washout, 4 weeks for fluoxetine).	OCD symptoms within 2 weeks; mean (SD) change in Y-BOCS scores_day 14_: I: −3.4 (6.1) (initial score: 28.6) vs. C3: −0.9 (5.0) (initial score: 27.0) (Placebo group allowed clomipramine IV after 2 weeks)
Faravelli *et al*., 1983 Italy	Double-blind RCT, double dummy	Inpatients, major depressive disorder, primary subtype, HRS: ≥18 after washout 5–7 days. I: *n* = 20 (13 women, mean (SD) age: 56.3 (9.8) years); C1: *n* = 20 (13 women, mean (SD) age: 58.2 (11.4) years).	I: Clomipramine IV (gradual titration, 0.5 g/kg every 3 days, maximum 2 mg/kg, maintained dose from day 10) C1: Clomipramine PO (gradual titration, 0.5 g/kg every 3 days, maximum 2 mg/kg, maintained dose from day 10). I and C1: Treatment duration: 4 weeks. Monotherapy (no other drugs allowed, apart from occasional lorazepam).	Depressive symptoms within 2 weeks; mean (SD) change in HRS scores: I: I: −11 (4.2)_day 7_, −14 (4.4)_day 14_ (initial score: 25) vs. C1: −7 (6.7)_day 7_, −11 (5.6)_day 14_ (initial score: 24) Long-term depressive symptoms; mean (SD) change in HRS scores_day 28_: I: −17 (5.5) (initial score: 25) vs. C1: −15 (6.3) (initial score: 24) Treatment discontinuation: I: 1/20 (cardiac arrhythmia during infusion) vs C1: 0/20 Adverse events related to administration via injection/infusion: I: 2/20 (one patient with known heart disease had cardiac arrhythmia during infusion, in week 4. One patient had thrombophlebitis that recovered spontaneously within 2 days) vs. C1: 0/20
Fähndrich, 1983 Germany	Open label RCT	Depression requiring medication, washout 4 days, after which sleep deprivation was carried out. I: *n* = 30 (24 women, mean (range) age: 49.9 (28–72) years); C2: *n* = 30 (21 women, mean (range) age: 54.6 (20–75) years).	I: Clomipramine IV (gradual titration, 75 mg/day for 3 days, subsequently 100 mg/day) C2: Maprotiline IV (gradual titration, 75 mg/day for 3 days, subsequently 100 mg/day). I and C2: Treatment duration: 3 weeks. Monotherapy (performed after washout).	Long-term depressive symptoms; response (≥50% decrease) in HRS scores_day 21_: I: 20/30 vs. C2: 21/30
Hordern *et al*., 1979 United Kingdom	Double-blind RCT, double dummy	*Part I*: Inpatients, primary depressive state. I: *n* = 20 (sex NR, 11/15 completers were women, mean (SD) age: 46 (15) years); C1: *n* = 15 (8/12 completers were women, mean (SD) age: 48 (15) years. *Part II*: Outpatients. I: *n* = 9 (7/8 completers were women, mean (SD) age: 44 (13) years; C1: *n* = 6 (2/4 completers were women, mean (SD) age: 49 (5) years).	I: Clomipramine IV (gradual titration, from 50 to 125 mg/day, four days a week) C1: Clomipramine PO (gradual titration, from 75 to 150 mg/day). I and C1: Treatment duration: 4 weeks. Monotherapy (diazepam, chlorpromazine, and nitrazepam kept to a minimum).	Depressive symptoms within 2 weeks; mean change in HRS scores: *Part I*: I: −10 (2.8)_week 1_, −13 (2.5)_week 2_ (initial score: 32) vs. C1: −11 (2.7)_week 1_, −13 (2.9)_week 2_ (initial score: 29); *Part II*: I: −8 (3.8)_week 1_, −14 (3.7)_week 2_ (initial score: 21) vs. C1: −6 (2.1)_week 1_, −11 (2.9)_week 2_ (initial score: 17) Long-term depressive symptoms; mean change in HRS scores_week 4_: *Part I*: I: −14 (2.8) (initial score: 32) vs. C1: −16 (3.0) (initial score: 29); *Part II*: I: −19 (3.4) (initial score: 21) vs. C1: −14 (3.1) (initial score: 17) Treatment discontinuation; *Part I*: I: 5/20 (three failed to improve, one hypotension, one collapse with vomiting after one infusion with an episode of hypotension 5 days later) vs. C1: 3/15 (two hypotension, on suspected jaundice which failed to develop); *Part II*: I: 1/9 (rapid deterioration) vs. C1: 2/6 (one failed to improve, one had thrombophlebitis) Adverse events related to administration via injection/infusion, *Part I*: I: 2/20 (both had hypotension, one of whom collapsed with vomiting and skin pallor at a prior infusion) vs. C1: 2/15 (both had hypotension); *Part II*: I: 0/9 vs. C1: 1/6 (thrombophlebitis)
Koran *et al*., 1997 United States	Double-blind RCT, double dummy	OCD, Y-BOCS ≥17. I: *n* = 7 (1 woman, mean (SD) age: 33.4 (4.3) years; C1: *n* = 8 (1 woman, mean (SD) age: 29.1 (4.3) years).	I: Clomipramine IV (pulse loaded, day 1: 150 mg, day 2: 200 mg, from 4.5 days after the second dose: 150 mg PO with increases of 25 mg/day to a maximum of 250 mg) C1: Clomipramine PO (pulse loaded, day 1: 150 mg, day 2: 200 mg, from 4.5 days after the second dose: 150 mg PO with increases of 25 mg/day to a maximum of 250 mg). I and C1: Treatment duration: 8 weeks. Trimethobenzamide administered before first dose.	OCD symptoms within 2 weeks; mean (SD) change in Y-BOCS scores: I: −11.0 (5.7)_week 1_, −10.2 (9.3)_week 2_ (initial score: 27.7) vs. C1: −0.9 (4.2)_week 1_, −1.7 (4.2)_week 2_ (initial score: 25.7) Long-term OCD symptoms_3 weeks_; mean (SD) change in Y-BOCS scores: I: −9.3 (11.4) (initial score: 27.2) vs. C1: −4.8 (9.8) (initial score: 26.2)
Koran *et al*., 2006 United States	Double-blind RCT, double dummy	OCD, Y-BOCS ≥20, failed 2 SRIs. I: *n* = 16; C1: *n* = 16. Sex in randomisation groups NR, in total 16 women. Age NR.	I: Clomipramine IV (pulse loaded, day 1: 150 mg, day 2: 200 mg, from day 6: 200 mg/day PO, increased to 250 mg/day if tolerated) C1: Clomipramine PO (pulse loaded, day 1: 150 mg, day 2: 200 mg, from day 6: 200 mg/day PO, increased to 250 mg/day if tolerated). I and C1: Treatment duration: 12 weeks. Monotherapy.	OCD symptoms within 2 weeks; mean (SD) change in Y-BOCS scores_day 6_: I: −2.75 (3.49) vs. C1: −4.38 (5.44) Long-term OCD symptoms; mean percent (SD) change in Y-BOCS scores_week 12_: I: 24 (26) vs. C1: 28 (27) (Absolute Y-BOCS scores NR) Treatment discontinuation: I: 3/16 (two due to insufficient response, one worsened depression) vs. C1: 5/16 (one each: consent, contact dermatitis, anxiety and depersonalisation, worsened depression, side effects) Adverse events related to administration via injection/infusion: I: 2/16 (treatment withheld due to bradycardia and tachycardia, respectively) vs. C: 2/16 (treatment withheld due to bradycardia and hypotension, respectively)
Lecrubier *et al*., 1980 France	Single-blind RCT, blinded raters	Inpatients with depressive syndrome (uni- and bipolar). I: *n* = 10 (6 women, mean age: 50.8 years); C2: *n* = 10 (6 women, mean age: 45.8 years).	I: Clomipramine IV (gradual titration, day 1: 75 mg, day 2: 100 mg, thereafter 150 mg/day) C2: Salbutamol IV (gradual titration, from 1.5 mg to 3 mg/infusion twice daily, i.e., 6 mg a day). I and C2: Treatment duration: 15 days. Monotherapy (diazepam, nitrazepam, levomepromazine allowed).	Depressive symptoms within 2 weeks; mean change in HRS scores_day5_: I: −4.35 (1.34) (initial score: 21.20) vs. C2: −-9.9 (1.94) (initial score: 23.25) Long-term depressive symptoms: mean change in HRS scores_day15_: I: −5.30 (1.72) (initial score: 21.2) vs. C2: −12.9 (2.8) (initial score: 23.25)
Pollock *et al*., 1989 United States	Double-blind RCT, double dummy	Inpatients with major depressive disorder (unipolar), HAM-D17 ≥15. Initial two-week drug-free period. I: *n* = 11 (10 women, mean (SD) age: 34 (7) years); C1: *n* = 11 (8 women, mean (SD) age: 42 (11) years).	I: Clomipramine IV (pulse loaded, day 1: 150 mg, day 2: 200 mg) C1: Clomipramine PO (pulse loaded, day 1: 150 mg, day 2: 200 mg). I and C1: Day 3–7: no treatment, day 8–28: clomipramine PO, 200 mg, adjustable. Monotherapy.	Depressive symptoms within 2 weeks; mean change in HAM-D_17_ scores: I: −9.8 (3.9)_day 7_, −12.9 (6.3)_day 14_ (initial score: 24.9) vs. C1: −11.4 (4.7)_day 7_, −13.5 (6.5)_day 14_ (initial score: 22.8) Long-term depressive symptoms; mean change in HAM-D_17_ scores: I: −14.7 (7.3) (initial score: 24.8) vs. C1: −15.7 (8.0) (initial score: 21.7) Treatment discontinuation: I: ? (one patient discontinued because of extreme anxiety before insertion of the butterfly cannula, randomisation group NR) vs. C1: 1+? (one because of nervousness; one patient, see I)
Sallee *et al*., 1997 United States	Double-blind RCT	Adolescents (14–18 years), outpatients, major depression. Initial 4-week washout for antidepressant (6 weeks for fluoxetine). I: *n* = 8 (2 women, mean (SD) age: 16.1 (0.9) years); C3: *n* = 8 (3 women, mean (SD) age: 16.4 (1.0) years).	I: Clomipramine IV (pulse load, day 1: 200 mg, no further infusions) C3: Saline placebo (day 1: infusion, not further infusions). Monotherapy.	Depressive symptoms within 2 weeks; mean change in HAM-D_21_ scores_day6_: I: −15 (4.1) (initial score: 22.9) vs. C3: −9.0 (6.1) (initial score: 22.4)
Spreux-Varoquaux *et al*., 1996 France	Double-blind RCT, double dummy	Nondeluded depressed inpatients (unipolar), MADRS ≥20. 7-day washout for antidepressants required. I: *n* = 13 (9 women, mean (SD) age: 50.5 (3.0) years); C1: *n* = 14 (11 women, mean (SD) age: 38.9 (2.2) years).	I: Clomipramine IV (gradual titration, from 25 to 75 mg/day, day 3–14: 75 mg/day) C1: Clomipramine PO (gradual titration, from 50 to 150 mg/day, day 3–14: 150mg/day). I and C1: Treatment duration: 14 days. Monotherapy (chlorzepate allowed).	Depressive symptoms within 2 weeks; mean (SD) change in MADRS scores_day 14_: I: −20.6 (15.6) (initial score: 34.8) vs. C1: −17.7 (11.4) (initial score: 32.9)

BPD, bipolar disorder; *C*, comparison; C1: clomipramine non-parenterally; C2: other treatment for the condition; C3: placebo; HADS, Hamilton depression scale; HAM-D_17_, Hamilton depression rating scale, 17 items; HAM-D_21_, Hamilton depression rating scale, 21 items; HDRS_17_, Hamilton depression rating scale, first 17 items; HDS, Hamilton depression scale; HRS, Hamilton rating scale; *I*, intervention; IM, intramuscular; IV, intravenous, MADRS, Montgomery-Åsberg depression rating scale, MDD, major depressive disorder, MDE, major depressive episode, NA, not applicable; NR, not reported; PO, per os; RCT, randomised controlled trial; SD, standard deviation; SRI, serotonin reuptake inhibitor; Y-BOCS, Yale-Brown obsessive- compulsive scale.

The intervention was intravenous (IV) clomipramine with gradual titration in eight RCTs (Escobar *et al*., 1973; Hordern *et al*., 1979; Lecrubier *et al*., 1980; de Cuyper *et al*., 1981; Fähndrich, 1983; Faravelli *et al*., 1983; Spreux-Varoquaux *et al*., 1996; Fallon *et al*., 1998), with pulse loading in four RCTs (Pollock *et al*., 1989; Koran *et al*., 1997, 2006; Sallee *et al*., 1997), and with a fixed dose in two RCTs (Drago *et al*., 1983; Altamura *et al*., 2008). The comparison was oral clomipramine (C1) in eight RCTs (Escobar *et al*., 1973; Hordern *et al*., 1979; de Cuyper *et al*., 1981; Faravelli *et al*., 1983; Pollock *et al*., 1989; Spreux-Varoquaux *et al*., 1996; Koran *et al*., 1997, 2006), another medication (C2) in four RCTs (Lecrubier *et al*., 1980; Drago *et al*., 1983; Fähndrich, 1983; Altamura *et al*., 2008), and placebo (C3) in two RCTs (Sallee *et al*., 1997; Fallon *et al*., 1998). ECT and ketamine were not used as a comparison in any RCT. Ten RCTs were assessed as not having a high risk of bias (Escobar *et al*., 1973; Hordern *et al*., 1979; Drago *et al*., 1983; Faravelli *et al*., 1983; Pollock *et al*., 1989; Spreux-Varoquaux *et al*., 1996; Koran *et al*., 1997, 2006; Sallee *et al*., 1997; Fallon *et al*., 1998). Reasons underlying the directness and risk of bias assessments are outlined in Table S2.

A summary of our findings, including reasons for downgrading according to GRADE, is presented in Table 2. No studies reported results for the outcomes suicide attempt, all-cause mortality, suicidal ideation, global functioning, length of hospital stay, or HRQL.

**Table 2.** Summary of findings for patients with depression and patients with OCD

Patients with depression				
Outcome	Comparison	Studies without high RoB (number of patients)	Result	Certainty of evidence
Depressive symptoms within 2 weeks	C1	5 RCTs (170 patients) *(whereof 3 were poolable)*	**No difference** Pooled change in HDRS scores, mean (SD): −1.27 (95% CI: −3.09; 0.54)	Low^1^
	C2	1 RCT (40 patients)	NA	Very low^2^
	C3	1 RCT (16 patients)	**Favours I** −6 (95% CI: −0.9; −11)	Low^3^
Depressive symptoms after more than two weeks	C1	4 RCT (143 patients) *(whereof 3 were poolable)*	**No difference** Pooled change in HDRS scores, mean (SD): −1.06 (95% CI: −4.70; 2.58)	Low^1^
	C2	2 RCT (83 patients)	NA	Very low^4^
Treatment discontinuation	C1	3 RCT (112 patients) *(whereof 2 were poolable)*	**No difference** Peto OR: 1.05 (95% CI: 0.30; 3.71)	Low^1^
	C2	1 RCT (36 patients)	0 vs 0 events	Very low^5^
Adverse events related to administration via injection/infusion	C1	3 RCT (121 patients)	NA	Very low^2^

Patients with OCD				
Outcome	Comparison	Studies without high RoB (number of patients)	Result	Certainty of evidence
OCD symptoms within 2 weeks	C1	2 RCT (47 patients)	NA	Very low^6^
	C3	1 RCT (54 patients)	**No difference** Change in Y-BOCS scores: −2.5 (95% CI: −5.6; 0.6)	Low^1^
OCD symptoms after more than two weeks	C1	1 RCT (15 patients)	NA	Very low^2^
Treatment discontinuation	C1	1 RCT (32 patients)	NA	Very low^2^
Adverse events related to administration via injection/infusion	C1	1 RCT (32 patients)	NA	Very low^2^

*C,* comparison; C1, oral clomipramine; C2, other treatment; C3, placebo; CI, confidence interval; HDRS, Hamilton depression rating scale; HRQL, health-related quality of life; *I*, intervention; NA, not applicable; OCD, obsessive-compulsive disorder.

1 Serious imprecision, some study limitations, some uncertainty regarding directness.

2 Very serious imprecision, some study limitations, some uncertainty regarding directness.

3 Serious indirectness, serious imprecision.

4 Serious study limitations, serious inconsistency, uncertain precision, some uncertainty regarding directness.

5 Serious study limitations, very serious indirectness, very serious imprecision.

6 Very serious inconsistency, serious imprecision, some study limitations, some uncertainty regarding directness.

### Patients with depression (P1)

Eleven RCTs, including a total of 317 patients, investigated parenteral clomipramine in patients with depression (Escobar *et al*., 1973; Hordern *et al*., 1979; Lecrubier *et al*., 1980; de Cuyper *et al*., 1981; Drago *et al*., 1983; Fähndrich, 1983; Faravelli *et al*., 1983; Pollock *et al*., 1989; Spreux-Varoquaux *et al*., 1996; Sallee *et al*., 1997; Altamura *et al*., 2008).

#### Depressive symptoms

For the comparison between parenteral and oral clomipramine (C1), six RCTs provided data regarding depressive symptoms within two weeks (Escobar *et al*., 1973; Hordern *et al*., 1979; de Cuyper *et al*., 1981; Faravelli *et al*., 1983; Pollock *et al*., 1989; Spreux-Varoquaux *et al*., 1996). Five double-blind RCTs were assessed not to have a high risk of bias and included a total of 170 patients (Escobar *et al*., 1973; Hordern *et al*., 1979; Faravelli *et al*., 1983; Pollock *et al*., 1989; Spreux-Varoquaux *et al*., 1996). Two of these could not be pooled; one did not report a summarised HDRS result (Escobar *et al*., 1973) and the other used MADRS (Spreux-Varoquaux *et al*., 1996). A forest plot of the changes in HDRS scores at two weeks for the remaining RCTs is presented in Figure 1A, with a pooled result of −1.27 (95% CI: −3.09 to 0.54; I2 = 22%). At one week, a meta-analysis of these studies revealed a mean difference of −0.71 (95% CI: −3.20 to 1.77; I2 = 62%). In the RCT reporting results using MADRS, the difference in change was −2.9 (95% CI: −13.3 to 7.5).

**Figure 2.**
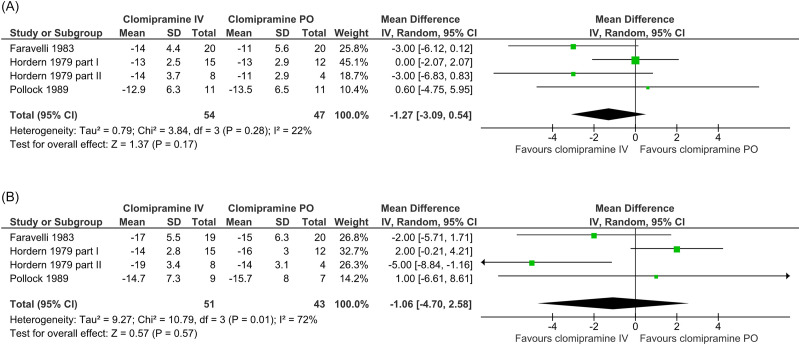
Forest plot and meta-analysis of changes in Hamilton depression rating scale (HDRS) scores at two (A) and four (B) weeks for the comparison intravenous (IV) versus oral (PO) clomipramine.

Five RCTs provided data regarding long-term depressive symptoms in patients with parenteral or oral clomipramine (Escobar *et al*., 1973; Hordern *et al*., 1979; de Cuyper *et al*., 1981; Faravelli *et al*., 1983; Pollock *et al*., 1989). Four double-blind RCTs were assessed not to have a high risk of bias and included a total of 143 patients(Escobar *et al*., 1973; Hordern *et al*., 1979; Faravelli *et al*., 1983; Pollock *et al*., 1989). One of these did not report a summarised HDRS result (Escobar *et al*., 1973). A meta-analysis of the changes in HDRS scores for the remaining RCTs is presented in Figure 2B, with a pooled result of −1.06 (95% CI: −4.70 to 2.58; I2 = 72%).

For the comparison between parenteral clomipramine and other treatments (C2), three RCTs contributed results regarding depressive symptoms within two weeks. The comparison was either maprotiline, citalopram (Altamura *et al*., 2008), or salbutamol (Lecrubier *et al*., 1980; Drago *et al*., 1983), all administered intravenously. One of them, comparing IV clomipramine with IV maprotiline, was assessed not to have a high risk of bias, included a total of 40 patients, and reported ‘no significant differences in the two groups’ (Drago *et al*., 1983).

Two RCTs contributed data regarding long-term depressive symptoms in patients with parenteral clomipramine or another treatment, either IV maprotiline (Fähndrich, 1983) or IV salbutamol (Lecrubier *et al*., 1980). Both were assessed to have a high risk of bias. The mean difference favoured IV salbutamol in one RCT: 7.6 (95% CI: 5.6 to 9.6) (Lecrubier *et al*., 1980). The other RCT reported similar response rates in both randomisation groups (Fähndrich, 1983).

For the comparison between parenteral clomipramine and placebo (C3), one RCT contributed results regarding depressive symptoms within two weeks (Sallee *et al*., 1997). It was assessed not to have a high risk of bias and included 16 adolescents. The reduction in HDRS scores at day 6 was larger in the intervention group, mean difference: −6.0 (95% CI: −0.9 to −11). No RCT contributed data regarding long-term depressive symptoms.

#### Treatment discontinuation

**Figure 3.**
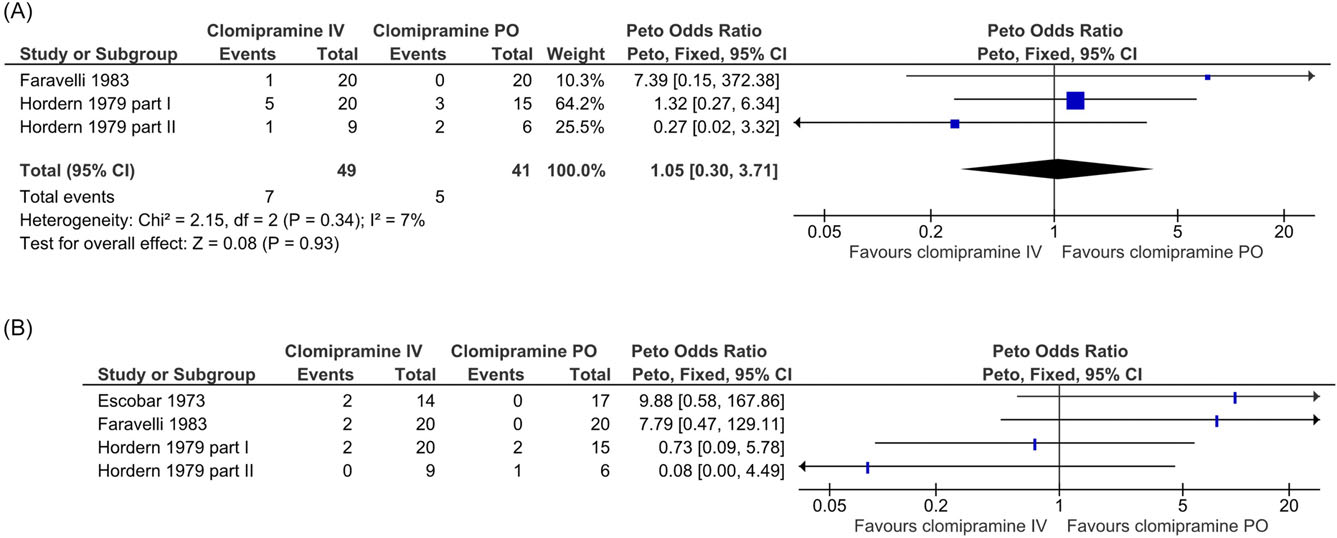
Forest plot and meta-analysis of treatment discontinuation (A) and forest plot of adverse events related to administration via injection/infusion (B; no meta-analysis was performed because of clinical heterogeneity of adverse events) for the comparison of intravenous (IV) versus oral (PO) clomipramine.

For the comparison between parenteral and oral clomipramine (C1), three double-blind RCTs contributed data regarding treatment discontinuation (Hordern *et al*., 1979; Faravelli *et al*., 1983; Pollock *et al*., 1989).They were all assessed not to have a high risk of bias and included a total of 112 patients. One RCT did not clearly report randomisation group allocation for one treatment discontinuation (Pollock *et al*., 1989) and could therefore not be pooled. A meta-analysis including the two RCTs providing poolable data is presented in Figure 3A, with a Peto odds ratio of 1.05 (95% CI: 0.30 to 3.71, I2 = 7%).

For the comparison between parenteral clomipramine and other treatments (C2), one RCT, using IV citalopram as the comparison and with a high risk of bias contributed the information that none of the 36 participants discontinued (Altamura *et al*., 2008).

No RCT contributed data regarding treatment discontinuation for the comparison between parenteral clomipramine and placebo (C3).

#### Adverse events related to administration via injection/infusion

For the comparison between parenteral and oral clomipramine (C1), three double-blind RCTs contributed data regarding infusion-related adverse events (Escobar *et al*., 1973; Hordern *et al*., 1979; Faravelli *et al*., 1983). They were all assessed not to have a high risk of bias and included a total of 121 patients. As all RCTs had a double dummy design, procedure-related adverse events could occur in both randomisation groups; one versus one event of thrombophlebitis occurred in these RCTs. In all, five versus two events related to the cardiovascular system occurred in the randomisation groups, whereof four versus two events were hypotension. These adverse events are in line with those described in the summary of product characteristics (SPC). A forest plot of all adverse events related to administration via injection/infusion is presented in Figure 3B. No meta-analysis was performed because of the clinical heterogeneity of the adverse events.

No RCT contributed data regarding infusion-related adverse events for the comparison between parenteral clomipramine and other treatments (C2) or placebo (C3),

### Patients with OCD (P2)

Three RCTs, including a total of 101 patients, investigated parenteral clomipramine in patients with OCD (Koran *et al*., 1997, 2006; Fallon *et al*., 1998).

#### OCD symptoms

**Figure 4.**
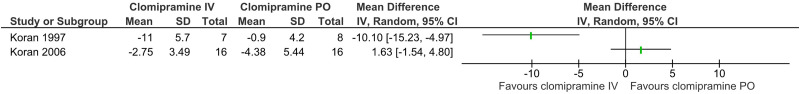
Forest plot of changes in Yale-Brown obsessive-compulsive scale (Y-BOCS) scores at one week for the comparison of intravenous (IV) versus oral (PO) clomipramine (no meta-analysis was performed because of clinical heterogeneity).

For the comparison between parenteral and oral clomipramine (C1), two double-blind RCTs contributed data regarding OCD symptoms within two weeks (Koran *et al*., 1997, 2006). They were both assessed not to have a high risk of bias and included a total of 47 patients. One RCT included patients who had previously not responded to serotonin reuptake inhibitors including clomipramine (Koran *et al*., 2006). A forest plot of changes in Y-BOCS scores at one week is presented in Figure 4. Because of clinical heterogeneity, we refrained from performing a meta-analysis.

One double-blind RCT provided data regarding long-term OCD symptoms in patients with parenteral or oral clomipramine (Koran *et al*., 1997). It was assessed not to have a high risk of bias, included 15 patients, and reported a non-significant change in Y-BOCS scores: −4.5 (95% CI: −16.5 to 7.5).

No RCT contributed data regarding OCD symptoms within two weeks or in the long-term for the comparison between parenteral clomipramine and other treatments (C2).

For the comparison between parenteral clomipramine and placebo (C3), one RCT contributed data regarding OCD symptoms within two weeks (Fallon *et al*., 1998). It was assessed not to have a high risk of bias, included 54 patients that were refractory to oral clomipramine, and reported no significant difference in Y-BOCS scores among those who completed the entire treatment (14 infusions): mean difference: −2.5 (95% CI: −5.6 to 0.6). Regarding long-term OCD symptoms, no RCT contributed data.

#### Treatment discontinuation

For the comparison between parenteral and oral clomipramine (C1), one double-blind RCT, contributed data regarding treatment discontinuation (Koran *et al*., 2006). It was assessed not to have a high risk of bias, included 32 patients, and reported three versus five events (out of 16 in each randomisation group).

No RCT contributed data regarding treatment discontinuation for the comparison between parenteral clomipramine and other treatments (C2) or placebo (C3).

#### Adverse events related to administration via injection/infusion

For the comparison between parenteral and oral clomipramine (C1), one double-blind RCT contributed data regarding infusion-related adverse events (Koran *et al*., 2006). It was assessed not to have a high risk of bias, included 32 patients, and reported that two patients in each randomisation group experienced adverse events related to the infusion. All events were related to the cardiovascular system.

No RCT contributed data regarding infusion-related adverse events for the comparison between parenteral clomipramine and other treatments (C2) or placebo (C3).

## Discussion

This systematic review shows that in patients with depression, parenteral clomipramine may not be superior to oral administration in terms of reducing depressive symptoms within two weeks. Given the MCID of 2 for HDRS (Hengartner and Plöderl, 2021) and as the upper confidence limit for symptom reduction exceeded 3, a clinically relevant effect of parenteral over oral clomipramine cannot be excluded. In the long term, there may be no difference. The same applies to treatment discontinuation rates. In patients with OCD overall, the evidence regarding potential differences in symptom alleviation between parenteral and oral clomipramine is too uncertain for conclusions. In the subgroup of patients with OCD refractory to oral clomipramine, parenteral clomipramine may not be superior to placebo, but a clinically relevant effect cannot be excluded. Across both conditions, data on key outcomes, including suicide attempts, all-cause mortality, suicidal ideation, global functioning, length of hospital stay, and HRQL, are lacking. Based on very low certainty of evidence, infusion-related adverse event rates reported in available RCTs are in line with the known safety profile.

Since low certainty evidence did not demonstrate benefits of parenteral compared to oral clomipramine in patients with depression, and infusion-related adverse events occurred, the benefit-risk balance may be unfavourable. A negative benefit-risk balance for parenteral clomipramine may also apply to patients with OCD, both in general and in those who have not responded to oral treatment. It is important to note that these results specifically pertain to parenteral administration, not to the substance clomipramine itself. In fact, both randomisation groups in the depression studies showed a clinically relevant reduction in depressive symptoms, regardless of how the medication was administered. However, clomipramine was ranked lower than other antidepressants in a comprehensive network analysis (Cipriani *et al*., 2018). A plausible explanation may be the lack of sedative and appetite-inducing effects of clomipramine, unlike medications such as amitriptyline and mirtazapine (Hieronymus *et al*., 2024). In the case of OCD, on the other hand, a clinically relevant effect on OCD symptoms was not consistent across the randomisation groups of the studies included in the current evidence synthesis.

One of the underlying arguments for parenteral instead of oral administration of clomipramine is to bypass the hepatic first-pass metabolism, which results in lower levels of its main metabolite, desmethylclomipramine. This may be relevant because clomipramine is a potent serotonin reuptake inhibitor, while its metabolite primarily inhibits noradrenaline reuptake (Gillman, 2007). However, a recent study reported that a higher ratio of desmethylclomipramine to clomipramine, in patients with depression, was more frequently observed in responders than non-responders (Vos *et al*., 2024). This finding suggests that the metabolism rationale for parenteral administration of clomipramine – namely, the purportedly more prominent serotonergic activity due to a higher parent-to-metabolite ratio – may not be applicable, consistent with our findings of no difference between parenteral and oral administration.

Many RCTs in the current evidence compilation were conducted during the 1980s. Their setting may thus not fully reflect current practices. Indeed, changes in the conceptualisation of depression have added complexity to comparing findings across different time periods (Parker *et al*., 2010; Ghaemi *et al*., 2012). The prevalence of severe depression, however, has remained relatively stable (Fugger *et al*., 2020) despite increased admission rates for mental, behavioural, and neurodevelopmental disorders (Naser *et al*., 2022). In the current systematic review, we intentionally used a broad definition in the PICO, including both unipolar and bipolar subtypes and without any age limits. This approach reflects real-world clinical practice, aiming to include the full spectrum of patients. However, since most RCTs included patients with severe depression, our findings may primarily be applicable to this subgroup.

For several outcomes selected for this systematic review due to their relevance for patients and healthcare, no data were available. For instance, results regarding the length of hospital stay could have added information, being a surrogate measure of short-term symptom reduction. Furthermore, no RCT studied the role of parenteral clomipramine as an alternative or adjunct to other rapid-acting interventions, such as ECT or ketamine, which are relevant in modern psychiatric care. Moving forward, more studies with relevant comparisons are warranted, particularly in depression, where the low certainty evidence does not rule out a short-term positive effect.

From a clinical decision-making perspective, our findings suggest that parenteral clomipramine should generally not be considered for the treatment of depression or OCD. The absence of clear short- or long-term advantages in symptom reduction in depression and the lack of convincing benefits over oral clomipramine or placebo in OCD, together with the infusion-related risks and resource requirements, make oral treatment preferable for most patients. Nevertheless, as a potential favourable effect cannot be entirely ruled out, parenteral clomipramine may be considered in highly selected cases, such as severely depressed inpatients who cannot reliably take oral medication or in whom rapid titration is more feasible via the IV route.

An important strength of this systematic review is that it provides a synthesis of currently available scientific evidence regarding the clinical value of parenteral clomipramine. Another major strength is the thorough and transparent performance, in line with the state-of-the art standards for this research design. Important limitations include that for both conditions studied – and particularly for OCD – the certainty of evidence was low or very low due to small sample sizes and few events. Further, our predefined primary outcome was symptom change within two weeks, which is shorter than the time frame typically required to observe full antidepressant or anti-obsessional effects. Moreover, the included RCTs had methodological shortcomings, such as unclear reporting of patient recruitment and randomisation procedure, the latter not uncommon at the time (Altman & Doré, 1990). Importantly, no RCT provided separate results for the HDRS ‘depressed mood’ item, which reportedly detects antidepressant effects more sensitively than the overall HDRS scores (Hieronymus *et al*., 2016). In addition, we pooled data from studies using different versions of the HDRS, an aspect that needs to be considered when pooled mean differences are interpreted. Similarly, the limitations associated with the Y-BOCS scale must be acknowledged (Storch *et al*., 2015; Cohen *et al*., 2024a), although it is a widely used tool for measuring longitudinal change. Finally, this systematic review does not address aspects like patient preferences, logistical and resource implications, or cost-effectiveness.

## Conclusion

The current evidence compilation shows that parenteral clomipramine may not be superior to oral administration in any respect, however, a short-term benefit in depression cannot be excluded.

## Supporting information

10.1017/neu.2026.10074.sm001 Ioannou et al. supplementary material Ioannou et al. supplementary material

## Data Availability

No additional data are available.

## Supplementary material

The supplementary material for this article can be found at https://doi.org/10.1017/neu.2026.10074.
