# Supplementary material for: Parenteral clomipramine for depression or obsessive-compulsive disorder: a systematic review and meta-analysis
Source: Acta Neuropsychiatr. 2026 Apr 14;38:e30. doi: 10.1017/neu.2026.10074 (PMC13130327; doi:10.1017/neu.2026.10074)
Supplement: Ioannou et al. supplementary material [file S092427082610074Xsup001.docx]

**Supplemental material**

Supplemental information to: Ioannou M, Falk Ö, Gustavsson J, Nilsson J, Sjögren P, Steingrimsson S, Svanberg T, Szabó Z, Wallerstedt SM. Parenteral Clomipramine for Depression or Obsessive-Compulsive Disorder: A Systematic Review and Meta-Analysis

**Table of content**

| Search strategies | | Page 2 |
| --- | --- | --- |
| Table S1 | Studies excluded after full-text reading, and reasons for exclusion | Page 7 |
| Table S2 | Reasons underlying study assessments of directness and risk of bias | Page 16 |
| Full references: Included studies & Excluded studies | | Page 19 |

**Search strategies**

**Database:** Ovid MEDLINE(R) ALL

**Date:** 30 sep 2024
**No. of results:** 2,060

| **Search** | **Query** | **Items found** |
| --- | --- | --- |
| 1 | exp Clomipramine/ | 2,854 |
| 2 | (chlorimipramine* or chlomipramine* or anafranil or hydiphen or clomipramine*).ab,kf,ti. | 3,707 |
| 3 | 1 or 2 | 4,441 |
| 4 | exp Mood Disorders/ | 175,487 |
| 5 | exp Depression/ | 161,947 |
| 6 | Compulsive Behavior/ | 3,553 |
| 7 | Obsessive Behavior/ | 1,377 |
| 8 | exp Obsessive-Compulsive Disorder/ | 18,198 |
| 9 | (depress* or antidepress* or anti depress* or bipolar or obsessive or compulsive or OCD).ab,kf,ti. | 723,436 |
| 10 | 4 or 5 or 6 or 7 or 8 or 9 | 781,746 |
| 11 | 3 and 10 | 3,156 |
| 12 | animals/ not (animals/ and humans/) | 5,228,303 |
| 13 | (animal or animals or rat or rats or mouse or mice or rodent or rodents or dog or dogs or cat or cats or cow or cows or hamster or hamsters or koalas or rabbit or rabbits or swine or murine or porcine or horses or horse or goats or goat or cadaver or cadaveric).ti. | 2,233,501 |
| 14 | 12 or 13 | 5,696,544 |
| 15 | 11 not 14 | 2,374 |
| 16 | limit 15 to (danish or english or norwegian or swedish) | 2,060 |

**Database: Embase** 1974 to 2024 September 27 (OvidSP)

**Date:** 30 sep 2024

**No. of results:** 3,300

| **#** | **Searches** | **Results** |
| --- | --- | --- |
| 1 | *clomipramine/ | 5,839 |
| 2 | (chlorimipramine* or chlomipramine* or anafranil or hydiphen or clomipramine*).ab,kf,ti. | 4,874 |
| 3 | 1 or 2 | 7,868 |
| 4 | exp mood disorder/ | 727,861 |
| 5 | exp obsessive compulsive disorder/ | 51,644 |
| 6 | (depress* or antidepress* or anti depress* or bipolar or obsessive or compulsive or OCD).ab,kf,ti. | 979,119 |
| 7 | 4 or 5 or 6 | 1,204,213 |
| 8 | 3 and 7 | 5,443 |
| 9 | animal/ not (animal/ and human/) | 1,230,833 |
| 10 | (animal or animals or rat or rats or mouse or mice or rodent or rodents or dog or dogs or cat or cats or cow or cows or hamster or hamsters or koalas or rabbit or rabbits or swine or murine or porcine or horses or horse or goats or goat or cadaver or cadaveric).ti. | 2,416,612 |
| 11 | 9 or 10 | 3,341,258 |
| 12 | 8 not 11 | 4,727 |
| 13 | limit 12 to (danish or english or norwegian or swedish) | 3,667 |
| 14 | limit 13 to (embase or medline) | 3,300 |

**Database:** The Cochrane Library
**Date:** 30 sep 2024
**No of results:** 658

*Cochrane reviews: 13
Cochrane protocols: 0
Trials: 645*

| **ID** | **Search** | **Hits** | |  |
| --- | --- | --- | --- | --- |
| #1 | MeSH descriptor: [Clomipramine] explode all trees | 485 | |  |
| #2 | (chlorimipramine* or chlomipramine* or anafranil or hydiphen or clomipramine*):ti,ab,kw (Word variations have been searched) | 1,015 | |  |
| #3 | #1 OR #2 | 1,015 | |  |
| #4 | MeSH descriptor: [Mood Disorders] explode all trees | 20,524 | |  |
| #5 | MeSH descriptor: [Depression] explode all trees | 18,683 | |  |
| #6 | MeSH descriptor: [Compulsive Behavior] explode all trees | 1,128 | |  |
| #7 | MeSH descriptor: [Obsessive Behavior] explode all trees | 65 | |  |
| #8 | MeSH descriptor: [Obsessive-Compulsive Disorder] explode all trees | 1,552 | |  |
| #9 | (depress* or antidepress* or (anti NEXT depress*) or bipolar or obsessive or compulsive or OCD):ti,ab,kw (Word variations have been searched) | 134,152 | |  |
| #10 | #4 OR #5 OR #6 OR #7 OR #8 OR #9 | 134,771 | |  |
| #11 | #3 AND #10 | 831 | |  |
| #12 | (clinicaltrials OR trialsearch):so | 535,215 | |  |
| #13 | (conference proceeding):pt | 248,848 | |  |
| #14 | #12 OR #13 | 784,063 | |  |
| #15 | #11 NOT #14 | 757 | |  |
| **Limit search to eng, dan, swe, nor** | | | **658** | |

___________________________________________________________________________

**Database:** PsycInfo

**Date:** 30 sep 2024

**No. of results:** 1,320

| **#** | **Query** | **Limiters/expanders** | **Results** |
| --- | --- | --- | --- |
| **S12** | **S8 NOT 9** | Expanders - Apply related words; Apply equivalent subjects Narrow by: Academic journals Narrow by Language: - swedish Narrow by Language: - english Search modes - Find all my search terms | **1,320** |
| S11 | S8 NOT S9 | Expanders - Apply related words; Apply equivalent subjects Narrow by Language: - swedish Narrow by Language: - english Search modes - Find all my search terms | 1,383 |
| S10 | S8 NOT S9 | Expanders - Apply related words; Apply equivalent subjects Search modes - Find all my search terms | 1,594 |
| S9 | TI (animal OR animals OR rat OR rats OR mouse OR mice OR rodent OR rodents OR dog OR dogs OR cat OR cats OR cow OR cows OR hamster OR hamsters OR koalas OR rabbit OR rabbits OR swine OR murine OR porcine OR horses or horse OR goats OR goat OR cadaver OR cadaveric) | Expanders - Apply related words; Apply equivalent subjects Search modes - Find all my search terms | 174,307 |
| S8 | S3 AND S7 | Expanders - Apply related words; Apply equivalent subjects Search modes - Find all my search terms | 1,817 |
| S7 | S4 OR S5 OR S6 | Expanders - Apply related words; Apply equivalent subjects Search modes - Find all my search terms | 452,525 |
| S6 | TI ( depress* or antidepress* or "anti depress*" or bipolar or obsessive or compulsive or OCD ) OR AB ( depress* or antidepress* or "anti depress*" or bipolar or obsessive or compulsive or OCD ) | Expanders - Apply related words; Apply equivalent subjects Search modes - Find all my search terms | 436,620 |
| S5 | DE "Obsessive Compulsive Disorder" OR DE "Body Dysmorphic Disorder" OR DE "Excoriation Disorder" OR DE "Hoarding Disorder" OR DE "Koro" OR DE "Trichotillomania" OR DE "Compulsions" OR DE "Repetition Compulsion" OR DE "Obsessions" OR DE "Obsessive Compulsive Personality Disorder" | Expanders - Apply related words; Apply equivalent subjects Search modes - Find all my search terms | 23,646 |
| S4 | DE "Major Depression" OR DE "Anaclitic Depression" OR DE "Dysthymic Disorder" OR DE "Endogenous Depression" OR DE "Late Life Depression" OR DE "Postpartum Depression" OR DE "Reactive Depression" OR DE "Recurrent Depression" OR DE "Seasonal Affective Disorder" OR DE "Treatment Resistant Depression" OR DE "Bipolar Disorder" OR DE "Bipolar I Disorder" OR DE "Bipolar II Disorder" OR DE "Cyclothymic Disorder" OR DE "Mania" OR DE "Bipolar I Disorder" OR DE "Bipolar II Disorder" OR DE "Depression (Emotion)" OR DE "Persistent Depressive Disorder" OR DE "Dysthymic Disorder" | Expanders - Apply related words; Apply equivalent subjects Search modes - Find all my search terms | 231,522 |
| S3 | S1 OR S2 | Expanders - Apply related words; Apply equivalent subjects Search modes - Find all my search terms | 2,136 |
| S2 | TI ( chlorimipramine* or chlomipramine* or anafranil or hydiphen or clomipramine* ) OR AB ( chlorimipramine* or chlomipramine* or anafranil or hydiphen or clomipramine* ) | Expanders - Apply related words; Apply equivalent subjects Search modes - Find all my search terms | 2,088 |
| S1 | DE "Chlorimipramine" | Expanders - Apply related words; Apply equivalent subjects Search modes - Find all my search terms | 1,163 |

**The websites listed below were visited on 25 Apr 2025.**

**Nothing relevant to the question at issue was found.**

| **toi** | **Search terms / Browsing** | **No. of results** | **No. of relevant results** |
| --- | --- | --- | --- |
| **SBU**  www.sbu.se ”Visa även träffar äldre än 5 år”, Sort: Rapporter | Klomipramin  Depression  Tvångssyndrom  OCD | 1  128  12  6 | 0  1  0  0 |
| **Folkehelseinstituttet (Norge)**  www.fhi.no | Category: Metodevurdering – Rapporter | 0 | 0 |
| **Behandlingsrådet (Danmark)**  https://behandlingsraadet.dk/ | Browsed | 0 | 0 |
| **Nationale Kliniske Anbefalinger og Retningslinjer (Danmark)**  https://www.sst.dk/da/Fagperson/Retningslinjer-og-procedurer/NKA-og-NKR/NKR-og-NKA-efter-omraade | Categories:  Mental sundhet Psykiske lidelser | 0 | 0 |
| **CAMTÖ**  https://www.regionorebrolan.se/sv/forskning/kontakt-och-organisation/hta-enheten-camto/ | Browsed | 0 | 0 |
| **HTA Region Stockholm**  https://www.chis.regionstockholm.se/hta/rapporter/ | Browsed | 0 | 0 |
| **Regional samverkansgrupp HTA (tidigare Metodrådet) i Sydöstra sjukvårdsregionen**  https://sydostrasjukvardsregionen.se/samverkansgrupper/hta/genomforda-bedomningar/ | Browsed | 0 | 0 |
| **HTA Syd**  https://vardgivare.skane.se/kompetens-utveckling/sakkunniggrupper/hta-skane/#110365 | Browsed | 0 | 0 |
| **Vetenskapliga rådet, Region Dalarna**  https://www.regiondalarna.se/plus/vard/utveckling-och-utbildning/kunskapsstyrning/vetenskapliga-radet/ | Browsed | 0 | 0 |

**Reference lists**

A citation search in Web of Science (both backwards and forwards) of included articles resulted in 752 references.

**Ongoing trials**

A search in Clinicaltrials.gov (4 March 2025) using the search terms (chlorimipramine OR chlomipramine OR anafranil OR hydiphen OR clomipramine) identified 37 trials. None of these fulfilled the PICO of this systematic review.

**Table S1.** Studies excluded after full-text reading, along with the reasons for their exclusion.

(For full citations, see pp 19‒29)

| **Author Year** | **Reason for exclusion** |
| --- | --- |
| Abu-Naser et al. 2021 | Wrong intervention: No mention of intravenous clomipramine |
| Ackerman et al. 2002 | Wrong intervention: No mention of intravenous clomipramine |
| Ackerman et al. 1996 | Wrong intervention: No mention of intravenous clomipramine |
| Ahmadpanah et al. 2017 | Wrong intervention: Buprenorphine |
| Alarcon et al. 1993 | Wrong study design: Case-series. No mention of intravenous clomipramine |
| Albert et al. 2018 | Systematic review with many interventions, intravenous clomipramine was mentioned briefly but no results |
| Alqdwah-Fattouh et al. 2020 | Wrong study design: Nested case-control. No mention of intravenous clomipramine. |
| Amin et al. 1977 | Wrong intervention: Oral administration |
| Amsterdam et al. 1997 | Wrong intervention: No mention of intravenous clomipramine |
| Ananth et al. 1977 | Wrong intervention: Oral administration |
| Bandelow et al. 2023 | Wrong publication type: Guideline |
| Beaumont et al. 1974 | Wrong publication type: Conference abstract |
| Bech et al. 1984 | Wrong intervention: No mention of intravenous clomipramine |
| Becker et al. 1971 | Wrong study design: Non-randomised study |
| Berman et al. 1995 | Wrong intervention: No mention of intravenous clomipramine |
| Bertolin et al. 2021 | Wrong study design: Systematic review (“intravenous” not mentioned) |
| Boaden et al. 2020 | Systematic review of systematic reviews: No mention of intravenous clomipramine |
| Buchholtz-Hansen et al. 1993 | Wrong intervention: No mention of intravenous clomipramine |
| Buoli et al. 2019 | Wrong study design: Non-randomised study |
| Burnand et al. 2002 | Wrong intervention: No mention of intravenous clomipramine |
| Carvajal Garcia-Pando et al. 2002 | Wrong study design, wrong intervention: No mention of intravenous clomipramine |
| Cassano et al. 1981 | Wrong intervention: Oral administration |
| Ceskova et al. 1981 | Wrong study design: Non-randomised study |
| Chistyakov et al. 2005 | Wrong intervention: No mention of intravenous clomipramine |
| Choi et al. 2009 | Wrong intervention: No mention of intravenous clomipramine |
| Christensen et al. 1985 | Wrong intervention: No mention of intravenous clomipramine |
| Cipriani et al. 2016 | Wrong intervention: No mention of intravenous clomipramine. Many different treatments |
| Cipriani et al. 2018 | Wrong intervention: No mention of intravenous clomipramine. Many different treatments |
| Civeira et al. 1990 | Wrong intervention: Oral administration |
| Cohen et al. 2024 | Wrong study design: Systematic review (“intravenous” not mentioned) |
| Collins et al. 1970 | Wrong study design: Case series |
| Collins et al. 1973 | Wrong study design: Non-randomised study |
| Cordes et al. 2009 | Wrong study design: Non-randomised study |
| de Oliveira et al. 2023 | Wrong publication type: Guideline |
| Degner et al. 2004 | Wrong publication type |
| Della Corte et al. 1979 | Wrong study design: Non-randomised study |
| Dencker et al. 1976 | Wrong study design: Non-randomised study |
| Desaunay et al. 2024 | Wrong study design: systematic review (“intravenous” not mentioned) |
| DeVeaugh-Geiss et al. 1992 | Wrong intervention: Oral administration |
| Dierick et al. 1990 | Wrong intervention: No mention of intravenous clomipramine |
| Dimitriou et al. 1984 | Wrong intervention: No mention of intravenous clomipramine |
| Diniz et al. 2010 | Wrong intervention: Oral administration |
| Eddy et al. 2004 | Wrong publication type: Metaanalysis. No mention of intravenous clomipramine |
| Ehlers et al. 1996 | Wrong study design: Case series |
| Elsenga et al. 1982 | Wrong intervention: No mention of intravenous clomipramine |
| Elsenga et al. 1987 | Wrong intervention: No mention of intravenous clomipramine |
| Erzegovesi et al. 2001 | Wrong intervention: Oral administration |
| Escobar et al. 1976 | Wrong study design: Case series |
| Escobar et al. 1977 | Wrong study design: Case series |
| Ewald et al. 1971 | Wrong study design: Case series |
| Faravelli et al. 1983b | Wrong study design: Non-randomised study |
| Faravelli et al. 1987a | Wrong publication type: Some kind of non-systematic review |
| Faravelli et al. 1987b | Wrong publication type, also duplicate |
| Farhat et al. 2020 | Systematic review with many interventions, no mention of intravenous clomipramine |
| Feng et al. 2007 | Wrong intervention: Oral administration |
| Feng et al. 2016 | Wrong intervention: Oral administration |
| Fineberg et al. 2007 | Wrong publication type: systematic review with no mention of intravenous clomipramine |
| Flament et al. 1985 | Wrong intervention: Oral administration |
| Fountain et al. 2020 | Wrong publication type, registry data, no mention of intravenous clomipramine |
| Friedrich et al. 2016 | Wrong publication type, registry data, no mention of intravenous clomipramine |
| Friedrich et al. 2022 | Wrong publication type, registry data, no mention of intravenous clomipramine |
| Fuglum et al. 1996 | Wrong intervention: No mention of intravenous clomipramine |
| Funke et al. 1990 | Wrong intervention: Oral administration |
| Fähndrich et al. 1983 | Wrong study design: Non-randomised study |
| Fähndrich et al. 1987 | Results according to our PICO presented in Fähndrich 1983 (included studies), no additional data presented |
| Geller et al. 2003 | Wrong publication type, no mention of intravenous clomipramine |
| Gentile et al. 2011 | Wrong publication type, no mention of intravenous clomipramine |
| Gex-Fabry et al. 1999 | Wrong study design: Case series |
| Golden et al. 1992 | Wrong study design: Non-randomised study |
| Golden et al. 2002 | Wrong study design: Non-randomised study |
| Gorenstein et al. 2006 | Wrong intervention: No mention of intravenous clomipramine |
| Grant et al. 2013 | Wrong study design: Non-randomised study. Wrong focus |
| Greil et al. 2019 | Wrong intervention: No mention of intravenous clomipramine |
| Greist et al. 1990 | Wrong intervention: No mention of intravenous clomipramine |
| Greist et al. 1995 | Too old (meta-analysis 1995). No mention of intravenous clomipramine |
| Grohmann et al. 1993 | Wrong intervention: No mention of intravenous clomipramine |
| Grohmann et al. 2004 | Wrong intervention: No mention of intravenous clomipramine |
| Guyotat et al. 1969 | Wrong language: French |
| Haghighi et al. 2013 | Wrong intervention: No mention of intravenous clomipramine |
| Hansen et al. 1994 | Wrong intervention: No mention of intravenous clomipramine |
| Hembree et al. 2003 | Wrong intervention: No mention of intravenous clomipramine |
| Hessov et al. 1969 | Wrong study design: Case series |
| Hewlett et al. 1992 | Wrong intervention: No mention of intravenous clomipramine |
| Hoehn-Saric et al. 1993 | Wrong intervention: No mention of intravenous clomipramine |
| Hoffman et al. 2021 | Wrong publication type: Review, no mention of intravenous clomipramine |
| Hojaij et al. 1995 | Wrong study design: Case series |
| Holper et al. 2020 | Meta-analysis with no mention of intravenous clomipramine |
| Hsu et al. 1995 | Wrong study design: Case series |
| Humble et al. 2001 | Wrong intervention: No mention of intravenous clomipramine |
| Humble et al. 2013 | Wrong intervention: No mention of intravenous clomipramine |
| Humble et al. 2016 | Wrong intervention: No mention of intravenous clomipramine |
| Insel et al. 1983a | Wrong intervention: D-amphetamine |
| Insel et al. 1983b | Wrong intervention: Oral administration |
| Jarrett et al. 1991 | Wrong study design: Case series |
| Jenike et al. 1989 | Wrong intervention: Oral administration |
| Jenike et al. 1990 | Old meta-analysis with no mention of intravenous clomipramine |
| Johnco et al. 2020 | Wrong publication type: Meta-analysis, no mention of intravenous clomipramine |
| Johnson et al. 1985 | Wrong intervention: No mention of intravenous clomipramine |
| Jouvent et al. 1998 | Wrong intervention: No mention of intravenous clomipramine |
| Joyce et al. 1994 | Wrong intervention: Oral administration |
| Jörgensen et al. 1984 | Wrong intervention: Oral administration |
| Karameh et al. 2015 | Wrong study design: Case series |
| Kasvikis et al. 1988a | Wrong intervention: Oral administration |
| Kasvikis et al. 1988b | Wrong intervention: No mention of intravenous clomipramine |
| Katz et al. 1990a | Wrong publication type, no mention of intravenous clomipramine |
| Katz et al. 1990b | Wrong intervention: No mention of intravenous clomipramine |
| Kessing et al. 2024 | Wrong intervention: No mention of intravenous clomipramine |
| Khan et al. 2004 | Wrong intervention: No mention of intravenous clomipramine |
| Khanna et al. 1988 | Wrong intervention: No mention of intravenous clomipramine |
| Klicpera et al. 1979 | Wrong study design: Non-randomised study |
| Klok et al. 1981 | Wrong intervention: Oral administration |
| Koran et al. 1998 | Wrong I/C: Pulse loading vs gradual dosing of intravenous clomipramine |
| Kornhaber et al. 1984 | Wrong intervention: Oral administration |
| Koszewska et al. 2009 | Wrong intervention, many different treatments, no mention of intravenous clomipramine |
| Kundermann et al. 2009 | Wrong intervention. Focus on sleep deprivation therapy. |
| Kupfer et al. 1994 | Wrong intervention: Oral administration |
| Kuss et al. 1986 | O missing: Focus on pharmacokinetics. Unclear randomisation |
| Landeros-Weisenberger et al. 2010 | Wrong intervention: No mention of intravenous clomipramine. Wrong publication type |
| Langer et al. 1983 | Wrong study design: Non-randomised study |
| Langer et al. 1984 | Wrong study design: Non-randomised study |
| Langer et al. 1986 | Wrong study design: Non-randomised study |
| Larisch et al. 2003 | Wrong P: Patients with depression in remission |
| Larsen et al. 1984 | Wrong study design: Case report |
| Lax et al. 1992 | Wrong intervention: No mention of intravenous clomipramine |
| Lechin et al. 1983 | Wrong intervention: No mention of intravenous clomipramine |
| Lejoyeux et al. 1993 | Wrong intervention: Oral administration |
| Leonard et al. 1995 | Wrong publication type, no mention of intravenous clomipramine |
| Leonard et al. 1988 | Wrong intervention: Oral administration |
| Leth-Moller et al. 2016 | Wrong study design, register study. No mention of intravenous clomipramine |
| Licht et al. 2013 | Wrong intervention: Oral administration |
| Limosin et al. 2006 | Wrong intervention: Oral administration |
| Linder et al. 1989 | Wrong intervention, oral administration |
| Lykouras et al. 2011 | Wrong study design: Non-randomised study |
| Ma et al. 2013 | Wrong intervention: No mention of intravenous clomipramine |
| Madalena et al. 1968 | Wrong language: Spanish |
| Maina et al. 2004 | Wrong study design: Non-controlled study |
| Mao et al. 2022 | Wrong study design: Systematic review (”intravenous” not mentioned) |
| Marazziti et al. 1997 | Wrong intervention: No mention of intravenous clomipramine |
| March et al. 1990 | Wrong intervention: Oral administration |
| Margat et al. 1969 | Wrong language: French |
| Marks et al. 1988 | Wrong intervention: Oral administration |
| Marshall et al. 1975 | Wrong study design: Case series |
| Mathew et al. 2001 | Wrong study design: Case series |
| Mavissakalian et al. 1985 | Wrong intervention: No mention of intravenous clomipramine |
| Mavissakalian et al. 1990 | Wrong intervention: No mention of intravenous clomipramine |
| Mawson et al. 1982 | Wrong intervention: No mention of intravenous clomipramine |
| McClure et al. 1973 | Wrong intervention: No mention of intravenous clomipramine |
| McGuire et al. 2014 | Wrong focus/Wrong intervention: Many different treatments, no mention of intravenous clomipramine |
| Merino et al. 2000 | Wrong intervention: No mention of intravenous clomipramine |
| Miccoli et al. 1978 | Wrong study design: Non-randomised study |
| Milanfranchi et al. 1997 | Wrong intervention: No mention of intravenous clomipramine |
| Miller et al. 1995 | Wrong intervention: No mention of intravenous clomipramine |
| Minelli et al. 2010 | O missing: Motor excitability |
| Monteiro et al. 1987 | Wrong intervention: Oral administration. |
| Montejo et al. 2001 | Wrong intervention: Many different treatments, no mention of intravenous clomipramine |
| Montgomery et al. 2001 | Wrong focus/Wrong study design |
| Moukaddam et al. 2004 | Wrong study design, no systematic review |
| Moyes et al. 1980 | Wrong intervention: No mention of intravenous clomipramine |
| Mumoli et al. 2014 | Wrong study design: Case-series. No treatment with intravenous clomipramine |
| Mundo et al. 1995 | C missing: All patients received intravenous clomipramine |
| Mundo et al. 1999 | C missing: All patients received intravenous clomipramine |
| Mundo et al. 2000 | Wrong intervention: Oral administration |
| Mundo et al. 1997 | Wrong intervention: Oral administration |
| Murphy et al. 1975 | Wrong intervention: Oral administration |
| Murphy et al. 1977 | Wrong intervention: Oral administration |
| Müller-Oerlinghausen et al. 1985 | Results according to our PICO presented in Fähndrich 1983 (included studies), no additional data presented |
| Möller et al. 1984 | Wrong intervention: Oral administration |
| Möller et al. 1990 | Wrong intervention: Oral administration |
| Nagayama et al. 1991 | Wrong intervention: Oral administration |
| Nahunek et al. 1984 | Wrong study design: Non-randomised study |
| Nielsen et al. 1990 | Wrong intervention: Oral administration |
| Ninan et al. 2000 | Wrong intervention: Oral administration |
| Noguera et al. 1991 | Wrong intervention: Oral administration |
| O'Flanagan et al. 1974 | Wrong publication type: Conference abstract |
| O'Sullivan et al. 1991 | Wrong intervention: Oral administration |
| Okayasu et al. 2012 | Wrong focus/Wrong intervention: No mention of intravenous clomipramine |
| Okayasu et al. 2019 | Wrong focus/Wrong intervention: No mention of intravenous clomipramine |
| Orgeta et al. 2017 | Wrong population: Alzheimer´s disease and depression. No mention of intravenous clomipramine |
| Pahus et al. 1970 | Wrong intervention: Oral administration |
| Pallanti et al. 1999 | Wrong intervention: Oral administration |
| Pandey et al. 2020 | Wrong intervention: Oral administration |
| Pato et al. 1988 | Wrong intervention: Oral administration |
| Pato et al. 1991 | Wrong intervention: Oral administration |
| Perroud et al. 2011 | Wrong intervention: No mention of intravenous clomipramine |
| Persson et al. 2007 | Wrong study design: Case series |
| Perugi et al. 2002 | Wrong focus/Wrong intervention: No mention of intravenous clomipramine |
| Pigott et al. 1990 | Wrong intervention: Oral administration |
| Pigott et al. 1991 | Wrong intervention: Oral administration |
| Pigott et al. 1992 | Wrong intervention: Oral administration |
| Pinder et al. 1980 | Wrong intervention: Oral administration |
| Pinkava et al. 1974 | Wrong study design: Non-randomised study |
| Pizarro et al. 2014 | Review with literature in only one database, no relevant studies on clomipramine |
| Pollock et al. 1993 | Wrong I/C: Pulse loading vs gradual dosing of intravenous clomipramine |
| Pollock et al. 1986 | Wrong study design: Case series |
| Porter et al. 2003 | Wrong intervention: No mention of intravenous clomipramine |
| Quilty et al. 2010 | Wrong intervention: No mention of intravenous clomipramine |
| Rabe-Jablonska et al. 2001 | Wrong comparison: Healthy volunteers. Wrong study design: non-randomised study |
| Rachman et al. 1979 | Wrong intervention: No mention of intravenous clomipramine |
| Rack et al. 1977 | Wrong study design: Case series |
| Rapisarda et al. 1982 | Wrong intervention: Oral administration. |
| Rapoport et al. 1980 | Wrong intervention: No mention of intravenous clomipramine |
| Ravizza et al. 1995 | Wrong intervention: No mention of intravenous clomipramine |
| Revet et al. 2020 | Wrong publication type, registry data, no mention of intravenous clomipramine |
| Riemann et al. 1990 | Wrong intervention: No mention of intravenous clomipramine |
| Ross et al. 2008 | Wrong study design: Case series |
| Rothbart et al. 2013 | Too old/Duplicate (See Hoffmann - same title) |
| Sallee et al. 1998a | Wrong study design: Randomised and non-randomised patients included, randomised patients not reported separately |
| Sallee et al. 1998b | Wrong study design: Non-randomised study |
| Sallee et al. 1989 | Wrong study design: Case series |
| Sanchez-Meca et al. 2014 | Systematic review with several different treatments, no mention of intravenous clomipramine |
| Scarzella et al. 1985 | Wrong intervention: No mention of intravenous clomipramine |
| Schlienger et al. 2004 | Wrong study design: Case-control. No mention of intravenous clomipramine |
| Schoretsanitis et al. 2020 | Wrong focus/Wrong intervention: Different antidepressants, no mention of intravenous clomipramine |
| Sepulveda-Lizcano et al. 2023 | Wrong focus/Wrong intervention: Different treatments, no mention of intravenous clomipramine |
| Serna et al. 2010 | Wrong focus/Wrong intervention: No mention of intravenous clomipramine |
| Shaw et al. 1975 | Wrong intervention: No mention of intravenous clomipramine |
| Silva et al. 1976 | Wrong intervention: Oral administration |
| Singer et al. 1968 | Wrong language: French |
| Skapinakis et al. 2016a | Wrong focus/Wrong intervention: Several different treatments |
| Skapinakis et al. 2016b | Wrong focus/Wrong intervention: Several different treatments |
| Skapinakis et al. 2021 | Reprint |
| Slikboer et al. 2017 | Wrong focus/Wrong intervention, several different treatments, no mention of intravenous clomipramine |
| Soomro et al. 2012 | Wrong focus/Wrong intervention, several different treatments, no mention of intravenous clomipramine |
| Souetre et al. 1996 | Wrong intervention: No mention of intravenous clomipramine |
| Souetre et al. 1997 | Wrong intervention: No mention of intravenous clomipramine |
| Statens beredning för medicinsk och social utvärdering (SBU) et al. 2004 | Several different treatments, no mention of intravenous clomipramine |
| Steinert et al. 2018 | Review with literature in only one database, no relevant studies on clomipramine |
| Stern et al. 1980 | Wrong intervention: No mention of intravenous clomipramine |
| Suchting et al. 2021 | Wrong focus/Wrong intervention: No mention of intravenous clomipramine |
| Swedo et al. 1989 | Wrong intervention: Oral administration |
| Szegedi et al. 1996 | Wrong intervention: No mention of intravenous clomipramine |
| Szymanska et al. 2001 | Wrong publication type: Non-randomised controlled trial |
| Tao et al. 2022 | Wrong focus/Wrong intervention: Several different treatments, no mention of intravenous clomipramine |
| Taylor et al. 2024 | Review with literature in only one database, no relevant studies on clomipramine |
| Thoren et al. 1980 | Wrong intervention: Oral administration |
| Uguz et al. 2019 | Wrong focus/Wrong intervention: Several different treatments, no mention of intravenous clomipramine |
| Uguz et al. 2021 | Wrong focus/Wrong intervention: Several different treatments, no mention of intravenous clomipramine |
| Ulrich et al. 1988 | Substudy of Fähndrich 1983 with EEG focus, no additional data according to our PICO presented |
| Ulrich et al. 1994 | O missing: EEG |
| van Kammen et al. 1980 | Wrong study design: Case series. Wrong comparison |
| van Scheyen et al. 1977 | Wrong intervention: Oral administration |
| Van Scheyen et al. 1979 | Wrong study design: No comparison of results |
| van Soest et al. 2007 | Wrong intervention: No mention of intravenous clomipramine |
| Varigonda et al. 2016 | Wrong focus/Wrong intervention: Several different treatments, no mention of intravenous clomipramine |
| Veale et al. 2014 | Wrong intervention: Several different treatments, no mention of intravenous clomipramine |
| Vencovsky et al. 1971 | Wrong study design: Case series |
| Vestergaard et al. 2008 | Wrong study design: Case-control. Wrong intervention, association between SSRIs and the risk of fracture, no mention of intravenous clomipramine |
| Viktorin et al. 2017 | Wrong intervention: Several different treatments, no mention of intravenous clomipramine |
| Voican et al. 2016 | Wrong focus/Wrong intervention: Several different treatments, no mention of intravenous clomipramine |
| Volavka et al. 1985 | Wrong intervention: Oral administration |
| Volmat et al. 1968 | Wrong language: French |
| Von Oefele et al. 1986 | Wrong study design. Wrong intervention: Tricyclic and MAOI therapy, no mention of intravenous clomipramine |
| Vos et al. 2023 | Wrong intervention: No mention of intravenous clomipramine |
| Warneke et al. 1992 | Wrong publication type: Comment |
| Waxman et al. 1977 | Wrong intervention: Oral administration |
| Wilson et al. 2004 | Wrong focus/Wrong intervention: Several different treatments, no mention of intravenous clomipramine |
| Winkler et al. 2021 | Wrong study design: Drug surveillance report, no mention of intravenous clomipramine |
| Wyndowe et al. 1975 | Wrong intervention: Oral administration |
| Wålinder et al. 1976 | Wrong intervention: No mention of intravenous clomipramine |
| Wålinder et al. 1981 | Wrong intervention: No clomipramine |
| Xiong et al. 2006 | Wrong focus: Several different treatments, no mention of intravenous clomipramine |
| Xu et al. 2021 | Wrong study design: Systematic review (”intravenous” not mentioned) |
| Yamada et al. 2003 | Wrong intervention: No mention of intravenous clomipramine |
| Younus et al. 2024 | Review with literature in only one database, no relevant studies on clomipramine |
| Zahn et al. 1984 | Wrong intervention: No mention of intravenous clomipramine |
| Zapletalek et al. 1982 | Wrong study design: Case series |
| Zhao et al. 1991 | Wrong language: Chinese |
| Zhou et al. 2024 | Systematic review, several different treatments, no mention of intravenous clomipramine |
| Zohar et al. 1988 | Wrong intervention: oral administration |
| Zohar et al. 1996 | Wrong intervention: oral administration |

C = comparison, EEG = electroencephalogram, I = intervention, MAOI = monoamine oxidase inhibitor, O = outcome, P = patients

**Table S2** Assessments of directness and risk of bias in included studies. The assessments apply to all outcomes if not explicitly stated otherwise.

(For full citations, see p 19)

| **Author Year** | **Problems contributing to downgrading the study in the assessment** | | | |
| --- | --- | --- | --- | --- |
|  | **Directness**^1^ | | **Risk of bias**^2^ | |
| Altamura  2008  Italy | - | Restricted to outpatients, i.e., a subgroup of the P  Patients with comorbidities were excluded. Patients with risk of suicide were excluded  Low doses of clomipramine (25 mg) and citalopram (10 mg)  No description or flowchart of participant recruitment  Characteristics NR | - | Randomisation process: High (randomisation procedure NR, characteristics in randomisation groups NR)  Effect of assignment to intervention: High (open-label, responders to SSRI and those dropping out because of side effects excluded)  Missing outcome data: Low  Outcome measurement: Some concerns (open-label, blinded raters)  Selection of reported results: Low |
| De Cuyper  1981  Belgium | ? | Restricted to outpatients, recently hospitalised, mean HDRS: 32 (20‒43)  3/10 were bipolar without mood stabilisers  No description or flowchart of participant recruitment | - | Randomisation process: High (randomisation procedure NR, older ages in C, 1 vs 2 patients with bipolar disorder)  Effect of assignment to intervention: High (open-label)  Missing outcome data: Low  Outcome measurement: High (Not blinded)  Selection of reported results: Low |
| Drago 1983  Italy | ? | C: maprotiline (not used today)  No description or flowchart of participant recruitment | +/? | Randomisation process: Some concerns (randomisation procedure NR, duration of symptoms: 220 vs. 157 days)  Effect of assignment to intervention: Low  Missing outcome data: Low  Outcome measurement: Low  Selection of reported results: Low |
| Escobar 1973 US | ? | No description or flowchart of participant recruitment | ? | Randomisation process: Some concerns (randomisation procedure NR, characteristics in randomisation groups not explicitly presented, reportedly 8/14 vs 4/17 had reactive depression, C “had slightly higher pathology scores”)  Effect of assignment to intervention: Low  Missing outcome data: Some concerns (dropouts replaced)  Outcome measurement: Low  Selection of reported results: Low |
| Fallon 1998 US | ? | P: no/partial response to clomipramine PO, or inadequate trial because of side effects  No description or flowchart of participant recruitment | ? | Randomisation process: Low  Effect of assignment to intervention: Some concerns (all patients were treatment refractory to oral clomipramine)  Missing outcome data: Low  Outcome measurement: Low  Selection of reported results: Low |
| Faravelli 1983 Italy | ? | No description or flowchart of participant recruitment | + | Randomisation process: Some concerns (randomisation procedure NR)  Effect of assignment to intervention: Low  Missing outcome data: Low  Outcome measurement: Low  Selection of reported results: Low |
| Fähndrich  1983  Germany | ? | Preceded by sleep deprivation  C: maprotiline (not used today)  No description or flowchart of participant recruitment | - | Randomisation process: Some concerns (randomisation procedure NR)  Effect of assignment to intervention: Some concerns (open-label but both groups received active treatment)  Missing outcome data: Low  Outcome measurement: High (open-label, assessments NR)  Selection of reported results: Low |
| Hordern 1979  UK | ? | Restricted to those who had not received ECT within three months and antidepressant drugs within two weeks  No description or flowchart of participant recruitment | ? | Randomisation process: Some concerns (randomisation procedure NR, only age and sex reported in comparison groups)  Effect of assignment to intervention: Low  Missing outcome data: Some concerns (8/35 dropped out in part I (6 vs. 2), 3/15 in part II (1 vs. 2))  Outcome measurement: Low  Selection of reported results: Low |
| Koran 1997  US | ? | No description or flowchart of participant recruitment | ? | Randomisation process: Some concerns (randomisation procedure NR)  Effect of assignment to intervention: Low  Missing outcome data: Some concerns (reasons for dropouts NR)  Outcome measurement: Low  Selection of reported results: Low |
| Koran 2006  US | ? | No flowchart of participant recruitment  Restricted to patients who had failed to benefit from two oral SRIs | ? | Randomisation process: Some concerns (characteristics in comparison groups only described as “no significant differences”, apart from age at onset: 18.7 vs. 13.1 years)  Effect of assignment to intervention: Some concerns (14/32 had not responded to clomipramine PO)  Missing outcome data: Low  Outcome measurement: Low  Selection of reported results: Low |
| Lecrubier 1980  France | - | C: salbutamol (not used)  No description or flowchart of participant recruitment | - | Randomisation process: Some concerns (used Taves procedures to allow equilibration, HRS score at baseline: 21.20 vs. 23.25, 3 vs. 6 patients had previous response to tricyclics treatment)  Effect of assignment to intervention: High (open-label)  Missing outcome data: Low  Outcome measurement: Some concerns (open-label, blinded raters)  Selection of reported results: Low |
| Pollock 1989  US | ? | No description or flowchart of participant recruitment | +/? | Randomisation process: Some concerns (randomisation procedure NR, 34 vs. 42 years)  Effect of assignment to intervention: Low  Missing outcome data: Low  Outcome measurement: Low  Selection of reported results: Low |
| Sallee 1997  US | - | No description or flowchart of participant recruitment  Restricted to adolescents, clomipramine PO not recommended to this age group according to Pharmaceutical Specialities in Sweden  Patients with risk of suicide were excluded  Restricted to patients with initial 4-week washout for antidepressant (6 weeks for fluoxetine) | + | Randomisation process: Low  Effect of assignment to intervention: Low  Missing outcome data: Low  Outcome measurement: Low  Selection of reported results: Low |
| Spreux-Varoquaux 1996  France | ? | P: No SSRI for 3 months required  No description or flowchart of participant recruitment | +/? | Randomisation process: Some concerns (randomisation procedure NR, 50 vs. 39 years)  Effect of assignment to intervention: Low  Missing outcome data: Low  Outcome measurement: Low  Selection of reported results: Low |

C = comparison, ECT = electroconvulsive treatment, EEG = electroencephalogram, HDRS = Hamilton depression rating scale, HRS = Hamilton rating scale, NR = not reported, PK = pharmacokinetics, PO = per os, SRI = serotonin reuptake inhibitor, SSRI = selective serotonin reuptake inhibitor, Y-BOCS = Yale-Brown obsessive compulsive scale

^1^Assessed using the checklist developed by the Centre for Health Technology Assessment, Sahlgrenska University Hospital, Region Västra Götaland, Gothenburg, Sweden: no/minor (+), some (?), or major (-) problems

^2^Assessed using the Cochrane risk-of-bias tool for randomised trials (RoB 2): low risk of bias (+), some concerns (?), or high risk of bias (-)

**Full references**

Included studies

1. Altamura AC, Dell'Osso B, Buoli M, Zanoni S, Mundo E. Intravenous augmentative citalopram versus clomipramine in partial/nonresponder depressed patients: a short-term, low dose, randomized, placebo-controlled study. J Clin Psychopharmacol. 2008;28(4):406-10. https://dx.doi.org/10.1097/JCP.0b013e31817d5931
2. de Cuyper HJ, van Praag HM, Mulder-Hajonides WR, Westenberg HG, de Zeeuw RA. Pharmacokinetics of clomipramine in depressive patients. Psychiatry Res. 1981;4(2):147-56. https://dx.doi.org/10.1016/0165-1781(81)90018-4
3. Drago F, Motta A, Grossi E. Intravenous maprotiline in severe and resistant primary depression: a double-blind comparison with clomipramine. J Int Med Res. 1983;11(2):78-84. https://dx.doi.org/10.1177/030006058301100203
4. Escobar JI, Flemenbaum A, Schiele BC. Chlorimipramine: a double-blind comparison of intravenous versus oral administration in depressed patients. Psychopharmacologia. 1973;33(2):111-6. https://dx.doi.org/10.1007/BF00429081
5. Fallon BA, Liebowitz MR, Campeas R, Schneier FR, Marshall R, Davies S, et al. Intravenous clomipramine for obsessive-compulsive disorder refractory to oral clomipramine: a placebo-controlled study. Arch Gen Psychiatry. 1998;55(10):918-24. https://dx.doi.org/10.1001/archpsyc.55.10.918
6. Faravelli C, Broadhurst AD, Ambonetti A, Ballerini A, De Biase L, La Malfa G, et al. Double-blind trial with oral versus intravenous clomipramine in primary depression. Biol Psychiatry. 1983a;18(6):695-706.
7. Fähndrich E. Effect of sleep deprivation as a predictor of treatment response to antidepressant medication. Acta Psychiatr Scand. 1983;68(5):341-4. https://dx.doi.org/10.1111/j.1600-0447.1983.tb07015.x
8. Hordern A, Seldrup J, Scient C. Intravenous clomipramine: Any real advantage? Journal of Pharmacotherapy. 1979;2:115-21.
9. Koran LM, Aboujaoude E, Ward H, Shapira NA, Sallee FR, Gamel N, et al. Pulse-loaded intravenous clomipramine in treatment-resistant obsessive-compulsive disorder. J Clin Psychopharmacol. 2006;26(1):79-83. https://dx.doi.org/10.1097/01.jcp.0000195112.24769.b3
10. Koran LM, Sallee FR, Pallanti S. Rapid benefit of intravenous pulse loading of clomipramine in obsessive-compulsive disorder. Am J Psychiatry. 1997;154(3):396-401. https://dx.doi.org/10.1176/ajp.154.3.396
11. Lecrubier Y, Puech AJ, Jouvent R, Simon P, Widlocher D. A beta adrenergic stimulant (salbutamol) versus clomipramine in depression: a controlled study. Br J Psychiatry. 1980;136:354-8. https://dx.doi.org/10.1192/bjp.136.4.354
12. Pollock BG, Perel JM, Nathan RS, Kupfer DJ. Acute antidepressant effect following pulse loading with intravenous and oral clomipramine. Arch Gen Psychiatry. 1989;46(1):29-35. https://dx.doi.org/10.1001/archpsyc.1989.01810010031005
13. Sallee FR, Vrindavanam NS, Deas-Nesmith D, Carson SW, Sethuraman G. Pulse intravenous clomipramine for depressed adolescents: double-blind, controlled trial. Am J Psychiatry. 1997;154(5):668-73. https://dx.doi.org/10.1176/ajp.154.5.668
14. Spreux-Varoquaux O, Gailledreau J, Vanier B, Bothua D, Plas J, Chevalier JF, et al. Initial increase of plasma serotonin: a biological predictor for the antidepressant response to clomipramine? Biol Psychiatry. 1996;40(6):465-73. https://dx.doi.org/10.1016/0006-3223(95)00449-1

Excluded studies

1. Abu-Naser D, Gharaibeh S, Al Meslamani AZ, Alefan Q, Abunaser R. Assessment of Extrapyramidal Symptoms Associated with Psychotropics Pharmacological Treatments, and Associated Risk Factors. Clin Pract Epidemol Ment Health. 2021;17:1-7. https://dx.doi.org/10.2174/1745017902117010001
2. Ackerman DL, Greenland S. Multivariate meta-analysis of controlled drug studies for obsessive-compulsive disorder. J Clin Psychopharmacol. 2002;22(3):309-17. https://dx.doi.org/10.1097/00004714-200206000-00012
3. Ackerman DL, Greenland S, Bystritsky A, Katz RJ. Relationship between early side effects and therapeutic effects of clomipramine therapy in obsessive-compulsive disorder. J Clin Psychopharmacol. 1996;16(4):324-8. https://dx.doi.org/10.1097/00004714-199608000-00009
4. Ahmadpanah M, Reihani A, Ghaleiha A, Soltanian A, Haghighi M, Jahangard L, et al. Buprenorphine augmentation improved symptoms of OCD, compared to placebo - Results from a randomized, double-blind and placebo-controlled clinical trial. J Psychiatr Res. 2017;94:23-8. https://dx.doi.org/10.1016/j.jpsychires.2017.06.004
5. Alarcon RD, Libb JW, Spitler D. A predictive study of obsessive-compulsive disorder response to clomipramine. J Clin Psychopharmacol. 1993;13(3):210-3.
6. Albert U, Marazziti D, Di Salvo G, Solia F, Rosso G, Maina G. A Systematic Review of Evidence-based Treatment Strategies for Obsessive- compulsive Disorder Resistant to first-line Pharmacotherapy. Curr Med Chem. 2018;25(41):5647-61. https://dx.doi.org/10.2174/0929867325666171222163645
7. Alqdwah-Fattouh R, Rodriguez-Martin S, de Abajo FJ, Gonzalez-Bermejo D, Gil M, Garcia-Lledo A, et al. Differential effects of antidepressant subgroups on risk of acute myocardial infarction: A nested case-control study. Br J Clin Pharmacol. 2020;86(10):2040-50. https://dx.doi.org/10.1111/bcp.14299
8. Amin MM, Ban TA, Pecknold JC, Klingner A. Clomipramine (Anafranil) and behaviour therapy in obsessive-compulsive and phobic disorders. J Int Med Res. 1977;5:33-7.
9. Amsterdam JD, Garcia-Espana F, Rosenzweig M. Clomipramine augmentation in treatment-resistant depression. Depress Anxiety. 1997;5(2):84-90.
10. Ananth J. Treatment of obsessive-compulsive neurosis with clomipramine (Anafranil). J Int Med Res. 1977;5:38-41.
11. Bandelow B, Allgulander C, Baldwin DS, Costa D, Denys D, Dilbaz N, et al. World Federation of Societies of Biological Psychiatry (WFSBP) guidelines for treatment of anxiety, obsessive-compulsive and posttraumatic stress disorders - Version 3. Part II: OCD and PTSD. World J Biol Psychiatry. 2023;24(2):118-34. https://dx.doi.org/10.1080/15622975.2022.2086296
12. Beaumont G, Gore CP. Aspects of a controlled, comparative trial of clomipramine (Anafranil), imipramine (Tofranil), electro convulsive therapy and a placebo. Journal de Pharmacologie. 1974;5(Sup. 2):5-6.
13. Bech P, Allerup P, Reisby N, Gram LF. Assessment of symptom change from improvement curves on the Hamilton depression scale in trials with antidepressants. Psychopharmacology (Berl). 1984;84(2):276-81. https://dx.doi.org/10.1007/BF00427459
14. Becker AL. A new adjunct to the treatment and management of depression: intravenous infusion of chlorimipramine (Anafranil). S Afr Med J. 1971;45(7):168-70.
15. Berman I, Sapers BL, Chang HH, Losonczy MF, Schmildler J, Green AI. Treatment of obsessive-compulsive symptoms in schizophrenic patients with clomipramine. J Clin Psychopharmacol. 1995;15(3):206-10. https://dx.doi.org/10.1097/00004714-199506000-00009
16. Bertolin S, Alonso P, Segalas C, Real E, Alemany-Navarro M, Soria V, et al. First manic/hypomanic episode in obsessive-compulsive disorder patients treated with antidepressants: A systematic review. J Psychiatr Res. 2021;137:319-27. https://dx.doi.org/10.1016/j.jpsychires.2021.02.060
17. Boaden K, Tomlinson A, Cortese S, Cipriani A. Antidepressants in Children and Adolescents: Meta-Review of Efficacy, Tolerability and Suicidality in Acute Treatment. Front Psychiatr. 2020;11:717. https://dx.doi.org/10.3389/fpsyt.2020.00717
18. Buchholtz-Hansen PE, Wang AG, Kragh-Sorensen P. Mortality in major affective disorder: relationship to subtype of depression. The Danish University Antidepressant Group. Acta Psychiatr Scand. 1993;87(5):329-35. https://dx.doi.org/10.1111/j.1600-0447.1993.tb03381.x
19. Buoli M, Rovera C, Pozzoli SM, Fiorentini A, Cremaschi L, Caldiroli A, et al. Is trazodone more effective than clomipramine in major depressed outpatients? A single-blind study with intravenous and oral administration. CNS Spectr. 2019;24(2):258-64. https://dx.doi.org/10.1017/S1092852917000773
20. Burnand Y, Andreoli A, Kolatte E, Venturini A, Rosset N. Psychodynamic psychotherapy and clomipramine in the treatment of major depression. Psychiatr Serv. 2002;53(5):585-90. https://dx.doi.org/10.1176/appi.ps.53.5.585
21. Carvajal Garcia-Pando A, Garcia del Pozo J, Sanchez AS, Velasco MA, Rueda de Castro AM, Lucena MI. Hepatotoxicity associated with the new antidepressants. J Clin Psychiatry. 2002;63(2):135-7. https://dx.doi.org/10.4088/jcp.v63n0208
22. Cassano GB, Castrogiovanni P, Mauri M, Rutigliano G, Pirro R, Cerone G, et al. A multicenter controlled trial in phobic-obsessive psychoneurosis. The effect of chlorimipramine and of its combinations with haloperidol and diazepam. Prog Neuropsychopharmacol. 1981;5(2):129-38. https://dx.doi.org/10.1016/0364-7722(81)90061-8
23. Ceskova E, Nahunek K, Rysanek R, Svestka J. Clinical experience with parenteral clomipramine and desipramine in the treatment of drug-resistant endogenous depression. Act Nerv Super (Praha). 1981;23(3):212-4.
24. Chistyakov AV, Kaplan B, Rubichek O, Kreinin I, Koren D, Feinsod M, et al. Antidepressant effects of different schedules of repetitive transcranial magnetic stimulation vs. clomipramine in patients with major depression: relationship to changes in cortical excitability. Int J Neuropsychopharmcol. 2005;8(2):223-33. https://dx.doi.org/10.1017/S1461145704004912
25. Choi YJ. Efficacy of treatments for patients with obsessive-compulsive disorder: a systematic review. J Am Acad Nurse Pract. 2009;21(4):207-13. https://dx.doi.org/10.1111/j.1745-7599.2009.00408.x
26. Christensen P, Thomsen HY, Pedersen OL, Gram LF, Kragh-Sorensen P. Orthostatic side effects of clomipramine and citalopram during treatment for depression. Psychopharmacology (Berl). 1985;86(4):383-5. https://dx.doi.org/10.1007/BF00427895
27. Cipriani A, Furukawa TA, Salanti G, Chaimani A, Atkinson LZ, Ogawa Y, et al. Comparative efficacy and acceptability of 21 antidepressant drugs for the acute treatment of adults with major depressive disorder: a systematic review and network meta-analysis. Lancet. 2018;391(10128):1357-66. https://dx.doi.org/10.1016/S0140-6736(17)32802-7
28. Cipriani A, Zhou X, Del Giovane C, Hetrick SE, Qin B, Whittington C, et al. Comparative efficacy and tolerability of antidepressants for major depressive disorder in children and adolescents: a network meta-analysis. Lancet. 2016;388(10047):881-90. https://dx.doi.org/10.1016/S0140-6736(16)30385-3
29. Civeira J, Cervera S, Giner J, Allen SR, Hellstern K, Malanowski H, et al. Moclobemide versus clomipramine in the treatment of depression: a multicentre trial in Spain. Acta Psychiatr Scand Suppl. 1990;360:48-9. https://dx.doi.org/10.1111/j.1600-0447.1990.tb05327.x
30. Cohen SE, Zantvoord JB, Storosum BWC, Mattila TK, Daams J, Wezenberg B, et al. Influence of study characteristics, methodological rigour and publication bias on efficacy of pharmacotherapy in obsessive-compulsive disorder: a systematic review and meta-analysis of randomised, placebo-controlled trials. BMJ Ment Health. 2024;27(1):12. https://dx.doi.org/10.1136/bmjment-2023-300951
31. Collins GH. Intravenous chlorimipramine in the treatment of severe depression. Br J Psychiatry. 1970;117(537):211-2.
32. Collins GH. The use of parenteral and oral chlorimipramine (Anafranil) in the treatment of depressive states. Br J Psychiatry. 1973;122(567):189-90. https://dx.doi.org/10.1192/bjp.122.2.189
33. Cordes J, Larisch R, Henning U, Thunker J, Werner C, Orozco G, et al. Abnormal neuroendocrine response to clomipramine in hereditary affective psychosis. Depress Anxiety. 2009;26(8):E111-9. https://dx.doi.org/10.1002/da.20405
34. de Oliveira MVS, de Barros PMF, de Mathis MA, Boavista R, Chacon P, Echevarria MAN, et al. Brazilian Research Consortium on Obsessive-Compulsive Spectrum Disorders guidelines for the treatment of adult obsessive-compulsive disorder. Part I: pharmacological treatment. Rev Bras Psiquiatr. 2023;45(2):146-61. https://dx.doi.org/10.47626/1516-4446-2022-2891
35. Degner D, Grohmann R, Kropp S, Ruther E, Bender S, Engel RR, et al. Severe adverse drug reactions of antidepressants: results of the German multicenter drug surveillance program AMSP. Pharmacopsychiatry. 2004;37:S39-45. https://dx.doi.org/10.1055/s-2004-815509
36. Della Corte L, Broadhurst AD, Sgaragli GP, Filippini S, Heeley AF, James HD, et al. Clinical response and tricyclic plasma levels during treatment with clomipramine. Br J Psychiatry. 1979;134:390-400. https://dx.doi.org/10.1192/bjp.134.4.390
37. Dencker SJ, Bake B. Investigation of the orthostatic reaction after intravenous administration of imipramine, chlorimipramine, and inimpramine-N-oxide. Acta Psychiatr Scand. 1976;54(1):74-8. https://dx.doi.org/10.1111/j.1600-0447.1976.tb00095.x
38. Desaunay P, Eslier M, Alexandre J, Dreyfus M, Chretien B, Guenole F. Antidepressants and fetal death: A systematic review and disproportionality analysis in the WHO safety database (VigiBase R). Psychiatry Res. 2024;339:116048. https://dx.doi.org/10.1016/j.psychres.2024.116048
39. DeVeaugh-Geiss J, Moroz G, Biederman J, Cantwell D, Fontaine R, Greist JH, et al. Clomipramine hydrochloride in childhood and adolescent obsessive-compulsive disorder--a multicenter trial. J Am Acad Child Adolesc Psychiatry. 1992;31(1):45-9. https://dx.doi.org/10.1097/00004583-199201000-00008
40. Dierick M, Cattiez P, Franck G, Burton P, Defleur J, Hermans W, et al. Moclobemide versus clomipramine in the treatment of depression: a double-blind multicentre study in Belgium. Acta Psychiatr Scand Suppl. 1990;360:50-1. https://dx.doi.org/10.1111/j.1600-0447.1990.tb05328.x
41. Dimitriou E, Paraschos A, Logothetis J. A double-blind comparison of lofepramine and clomipramine in depressed outpatients. Psychopharmacol Bull. 1984;20(4):684-7.
42. Diniz JB, Shavitt RG, Pereira CA, Hounie AG, Pimentel I, Koran LM, et al. Quetiapine versus clomipramine in the augmentation of selective serotonin reuptake inhibitors for the treatment of obsessive-compulsive disorder: a randomized, open-label trial. J Psychopharmacol. 2010;24(3):297-307. https://dx.doi.org/10.1177/0269881108099423
43. Eddy KT, Dutra L, Bradley R, Westen D. A multidimensional meta-analysis of psychotherapy and pharmacotherapy for obsessive-compulsive disorder. Clin Psychol Rev. 2004;24(8):1011-30. https://dx.doi.org/10.1016/j.cpr.2004.08.004
44. Ehlers CL, Havstad JW, Kupfer DJ. Estimation of the time course of slow-wave sleep over the night in depressed patients: effects of clomipramine and clinical response. Biol Psychiatry. 1996;39(3):171-81. https://dx.doi.org/10.1016/0006-3223(95)00139-5
45. Elsenga S, van den Hoofdakker RH. Clinical effects of sleep deprivation and clomipramine in endogenous depression. J Psychiatr Res. 1982;17(4):361-74. https://dx.doi.org/10.1016/0022-3956(82)90041-3
46. Elsenga S, Van den Hoofdakker RH. Response to total sleep deprivation and clomipramine in endogenous depression. J Psychiatr Res. 1987;21(2):151-61. https://dx.doi.org/10.1016/0022-3956(87)90015-x
47. Erzegovesi S, Cavallini MC, Cavedini P, Diaferia G, Locatelli M, Bellodi L. Clinical predictors of drug response in obsessive-compulsive disorder. J Clin Psychopharmacol. 2001;21(5):488-92. https://dx.doi.org/10.1097/00004714-200110000-00006
48. Escobar JI, Gomez O, Tuason VB. Depressive subtypes, blood pressure changes and response to treatment. Dis Nerv Syst. 1977;38(2):76-9.
49. Escobar JI, Teeter RR, Tuason VB, Schiele BC. Intravenous chlorimipramine and depressive subtypes. Dis Nerv Syst. 1976;37(6):325-8.
50. Ewald G, Och GP, Werne-Lindenberg R. Chlorimipramine infusions. A new alternative in depression therapy. Nord Psykiatr Tidsskr. 1971;25(3):247-52. https://dx.doi.org/10.3109/08039487109094664
51. Faravelli C, Brat A, Marchetti G, Franchi F, Padeletti L, Michelucci A, et al. Cardiac effects of clomipramine treatment. ECG and left ventricular systolic time intervals. Neuropsychobiology. 1983b;9(2):113-8. https://dx.doi.org/10.1159/000117948
52. Faravelli C, Pallanti S. Clomipramine by different routes of administration: short- and long-term efficacy and predictors of clinical outcome. Psychopharmacol Bull. 1987b;23(3):459-63.
53. Faravelli C, Pallanti S. Antidepressant drugs: Onset and therapeutic specificity of action. Clomipramine by different routes of administration: Short- and long-term efficacy and predictors of clinical outcome. Psychopharmacol Bull. 1987a;23(3):459-63.
54. Farhat LC, Olfson E, Nasir M, Levine JLS, Li F, Miguel EC, et al. Pharmacological and behavioral treatment for trichotillomania: An updated systematic review with meta-analysis. Depress Anxiety. 2020;37(8):715-27. https://dx.doi.org/10.1002/da.23028
55. Feng B, Liu L, Fangzhong X, Chen J, Wang P, Chen W, et al. Thirty cases of obsession treated by point-stimulation and with small dose of chlorimipramine. J Tradit Chin Med. 2007;27(1):3-6.
56. Feng B, Zhang ZJ, Zhu RM, Yuan GZ, Luo LY, McAlonan GM, et al. Transcutaneous electrical acupoint stimulation as an adjunct therapy for obsessive-compulsive disorder: A randomized controlled study. J Psychiatr Res. 2016;80:30-7. https://dx.doi.org/10.1016/j.jpsychires.2016.05.015
57. Fineberg NA, Pampaloni I, Pallanti S, Ipser J, Stein DJ. Sustained response versus relapse: the pharmacotherapeutic goal for obsessive-compulsive disorder. Int Clin Psychopharmacol. 2007;22(6):313-22. https://dx.doi.org/10.1097/YIC.0b013e32825ea312
58. Flament MF, Rapoport JL, Kilts C. A controlled trial of clomipramine in childhood obsessive compulsive disorder. Psychopharmacol Bull. 1985;21(1):150-2.
59. Fountain JS, Tomlin AM, Reith DM, Tilyard MW. Fatal Toxicity Indices for Medicine-Related Deaths in New Zealand, 2008-2013. Drug Saf. 2020;43(3):223-32. https://dx.doi.org/10.1007/s40264-019-00885-4
60. Friedrich ME, Akimova E, Huf W, Konstantinidis A, Papageorgiou K, Winkler D, et al. Drug-Induced Liver Injury during Antidepressant Treatment: Results of AMSP, a Drug Surveillance Program. Int J Neuropsychopharmcol. 2016;19(4) https://dx.doi.org/10.1093/ijnp/pyv126
61. Friedrich ME, Grohmann R, Rabl U, Winkler D, Konstantinidis A, Engel R, et al. Incidence of Drug-Induced Delirium During Treatment With Antidepressants or Antipsychotics: A Drug Surveillance Report of German-Speaking Countries Between 1993 and 2016. Int J Neuropsychopharmcol. 2022;25(7):556-66. https://dx.doi.org/10.1093/ijnp/pyac005
62. Fuglum E, Rosenberg C, Damsbo N, Stage K, Lauritzen L, Bech P. Screening and treating depressed patients. A comparison of two controlled citalopram trials across treatment settings: hospitalized patients vs. patients treated by their family doctors. Danish University Antidepressant Group. Acta Psychiatr Scand. 1996;94(1):18-25. https://dx.doi.org/10.1111/j.1600-0447.1996.tb09819.x
63. Funke HJ, Moritz E, Hellstern K, Malanowski H. Moclobemide versus clomipramine in the treatment of depression: a single-centre study, Federal Republic of Germany. Acta Psychiatr Scand Suppl. 1990;360:46-7. https://dx.doi.org/10.1111/j.1600-0447.1990.tb05326.x
64. Fähndrich E. Clinical and biological parameters as predictors for antidepressant drug responses in depressed patients. Pharmacopsychiatria. 1983;16(6):179-85. https://dx.doi.org/10.1055/s-2007-1019495
65. Fähndrich E. Biological predictors of success of antidepressant drug therapy. Psychiatr Dev. 1987;5(2):151-71.
66. Geller DA, Biederman J, Stewart SE, Mullin B, Martin A, Spencer T, et al. Which SSRI? A meta-analysis of pharmacotherapy trials in pediatric obsessive-compulsive disorder. Am J Psychiatry. 2003;160(11):1919-28. https://dx.doi.org/10.1176/appi.ajp.160.11.1919
67. Gentile S. Efficacy of antidepressant medications in children and adolescents with obsessive-compulsive disorder: a systematic appraisal. J Clin Psychopharmacol. 2011;31(5):625-32. https://dx.doi.org/10.1097/JCP.0b013e31822bb1ff
68. Gex-Fabry M, Balant-Gorgia AE, Balant LP. Clomipramine concentration as a predictor of delayed response: a naturalistic study. Eur J Clin Pharmacol. 1999;54(12):895-902. https://dx.doi.org/10.1007/s002280050572
69. Golden RN, Ekstrom D, Brown TM, Ruegg R, Evans DL, Haggerty JJ, Jr., et al. Neuroendocrine effects of intravenous clomipramine in depressed patients and healthy subjects. Am J Psychiatry. 1992;149(9):1168-75. https://dx.doi.org/10.1176/ajp.149.9.1168
70. Golden RN, Heine AD, Ekstrom RD, Bebchuk JM, Leatherman ME, Garbutt JC. A longitudinal study of serotonergic function in depression. Neuropsychopharmacology. 2002;26(5):653-9. https://dx.doi.org/10.1016/S0893-133X(01)00406-7
71. Gorenstein C, de Carvalho SC, Artes R, Moreno RA, Marcourakis T. Cognitive performance in depressed patients after chronic use of antidepressants. Psychopharmacology (Berl). 2006;185(1):84-92. https://dx.doi.org/10.1007/s00213-005-0274-2
72. Grant JE, Mancebo MC, Weinhandl E, Odlaug BL, Eisen JL, Rasmussen SA. Longitudinal course of pharmacotherapy in obsessive-compulsive disorder. Int Clin Psychopharmacol. 2013;28(4):200-5. https://dx.doi.org/10.1097/YIC.0b013e3283613e4d
73. Greil W, Zhang X, Stassen H, Grohmann R, Bridler R, Hasler G, et al. Cutaneous adverse drug reactions to psychotropic drugs and their risk factors - a case-control study. Eur Neuropsychopharmacol. 2019;29(1):111-21. https://dx.doi.org/10.1016/j.euroneuro.2018.10.010
74. Greist JH, Jefferson JW, Kobak KA, Katzelnick DJ, Serlin RC. Efficacy and tolerability of serotonin transport inhibitors in obsessive-compulsive disorder. A meta-analysis. Arch Gen Psychiatry. 1995;52(1):53-60. https://dx.doi.org/10.1001/archpsyc.1995.03950130053006
75. Greist JH, Jefferson JW, Rosenfeld R, Gutzmann LD, March JS, Barklage NE. Clomipramine and obsessive compulsive disorder: a placebo-controlled double-blind study of 32 patients. J Clin Psychiatry. 1990;51(7):292-7.
76. Grohmann R, Hippius H, Helmchen H, Ruther E, Schmidt LG. The AMUP study for drug surveillance in psychiatry - a summary of inpatient data. Pharmacopsychiatry. 2004;37:S16-26. https://dx.doi.org/10.1055/s-2004-815507
77. Grohmann R, Strobel C, Ruther E, Dirsched P, Helmchen H, Hippius H, et al. Adverse psychic reactions to psychotropic drugs - A report from the AMUP study. Pharmacopsychiatry. 1993;26:84-93. http://dx.doi.org/10.1055/s-2007-1014348
78. Guyotat J. Classic indications for anafranil. L'Encéphale: Revue de psychiatrie clinique biologique et thérapeutique. 1969;58:61-6.
79. Haghighi M, Jahangard L, Mohammad-Beigi H, Bajoghli H, Hafezian H, Rahimi A, et al. In a double-blind, randomized and placebo-controlled trial, adjuvant memantine improved symptoms in inpatients suffering from refractory obsessive-compulsive disorders (OCD). Psychopharmacology (Berl). 2013;228(4):633-40. https://dx.doi.org/10.1007/s00213-013-3067-z
80. Hansen PE, Wang AG, Kragh-Sorensen P. Mortality--suicide and natural death--among depressed patients. Relation to type of depression. Ugeskr Laeger. 1994;156(48):7224-8.
81. Hembree EA, Riggs DS, Kozak MJ, Franklin ME, Foa EB. Long-term efficacy of exposure and ritual prevention therapy and serotonergic medications for obsessive-compulsive disorder. CNS Spectr. 2003;8(5):363-71, 81. https://dx.doi.org/10.1017/s1092852900018629
82. Hessov I. Blood pressure changes in treatment with Anafranil. Nord Psykiatr Tidsskr. 1969;23(3):233-6. https://dx.doi.org/10.3109/08039486909103678
83. Hewlett WA, Vinogradov S, Agras WS. Clomipramine, clonazepam, and clonidine treatment of obsessive-compulsive disorder. J Clin Psychopharmacol. 1992;12(6):420-30.
84. Hoehn-Saric R, McLeod DR, Zimmerli WD, Hipsley PA. Symptoms and physiologic manifestations in obsessive compulsive patients before and after treatment with clomipramine. J Clin Psychiatry. 1993;54(7):272-6.
85. Hoffman J, Williams T, Rothbart R, Ipser JC, Fineberg N, Chamberlain SR, et al. Pharmacotherapy for trichotillomania. Cochrane Database Syst Rev. 2021;9:CD007662. https://dx.doi.org/10.1002/14651858.CD007662.pub3
86. Hojaij CR. Reconsidering antidepressants infusions for resistant depressions in out-patient service. Neurology Psychiatry and Brain Research. 1995;3:129-32.
87. Holper L. Optimal doses of antidepressants in dependence on age: Combined covariate actions in Bayesian network meta-analysis. EClinicalMedicine. 2020;18:100219. https://dx.doi.org/10.1016/j.eclinm.2019.11.012
88. Hsu JH, Shen WW. Male sexual side effects associated with antidepressants: a descriptive clinical study of 32 patients. Int J Psychiatry Med. 1995;25(2):191-201. https://dx.doi.org/10.2190/1DHU-Y7L7-9GKG-V7WV
89. Humble M, Bejerot S, Bergqvist PB, Bengtsson F. Reactivity of serotonin in whole blood: relationship with drug response in obsessive-compulsive disorder. Biol Psychiatry. 2001;49(4):360-8. https://dx.doi.org/10.1016/s0006-3223(00)00956-2
90. Humble MB, Bejerot S. Orgasm, Serotonin Reuptake Inhibition, and Plasma Oxytocin in Obsessive-Compulsive Disorder. Gleaning From a Distant Randomized Clinical Trial. Sex. 2016;4(3):e145-55. https://dx.doi.org/10.1016/j.esxm.2016.04.002
91. Humble MB, Uvnas-Moberg K, Engstrom I, Bejerot S. Plasma oxytocin changes and anti-obsessive response during serotonin reuptake inhibitor treatment: a placebo controlled study. BMC Psychiatry. 2013;13:344. https://dx.doi.org/10.1186/1471-244X-13-344
92. Insel TR, Hamilton JA, Guttmacher LB, Murphy DL. D-amphetamine in obsessive-compulsive disorder. Psychopharmacology (Berl). 1983a;80(3):231-5. https://dx.doi.org/10.1007/BF00436159
93. Insel TR, Murphy DL, Cohen RM, Alterman I, Kilts C, Linnoila M. Obsessive-compulsive disorder. A double-blind trial of clomipramine and clorgyline. Arch Gen Psychiatry. 1983b;40(6):605-12. https://dx.doi.org/10.1001/archpsyc.1983.04390010015002
94. Jarrett DB, Pollock B, Miewald JM, Kupfer DJ. Acute effect of intravenous clomipramine upon sleep-related hormone secretion in depressed outpatients and healthy control subjects. Biol Psychiatry. 1991;29(1):3-14. https://dx.doi.org/10.1016/0006-3223(91)90206-2
95. Jenike MA, Baer L, Greist JH. Clomipramine versus fluoxetine in obsessive-compulsive disorder: a retrospective comparison of side effects and efficacy. J Clin Psychopharmacol. 1990;10(2):122-4. https://dx.doi.org/10.1097/00004714-199004000-00008
96. Jenike MA, Baer L, Summergrad P, Weilburg JB, Holland A, Seymour R. Obsessive-compulsive disorder: a double-blind, placebo-controlled trial of clomipramine in 27 patients. Am J Psychiatry. 1989;146(10):1328-30. https://dx.doi.org/10.1176/ajp.146.10.1328
97. Johnco C, McGuire JF, Roper T, Storch EA. A meta-analysis of dropout rates from exposure with response prevention and pharmacological treatment for youth with obsessive compulsive disorder. Depress Anxiety. 2020;37(5):407-17. https://dx.doi.org/10.1002/da.22978
98. Johnson BG. En dubbel-blind jämförelse mellan Amoxapin och Clomipramin = A double-blind comparative study of amoxapine and clomipramine. Nord Psykiatr Tidsskr. 1985;39(5):405-8. https://dx.doi.org/10.3109/08039488509101929
99. Jouvent R, Le Houezec J, Payan C, Mikkelsen H, Fermanian J, Millet V, et al. Dimensional assessment of onset of action of antidepressants: a comparative study of moclobemide vs. clomipramine in depressed patients with blunted affect and psychomotor retardation. Psychiatry Res. 1998;79(3):267-75. https://dx.doi.org/10.1016/s0165-1781(98)00046-8
100. Joyce PR, Mulder RT, Cloninger CR. Temperament predicts clomipramine and desipramine response in major depression. J Affect Disord. 1994;30(1):35-46. https://dx.doi.org/10.1016/0165-0327(94)90149-x
101. Jörgensen B, Norrelund N, Bech P, Jakobsen K. Pain and depressive symptoms in general practice. A controlled study of Anafranil (clomipramine) versus placebo. Ugeskr Laeger. 1984;146(38):2868-72.
102. Karameh WK, Khani M. Intravenous Clomipramine for Treatment-Resistant Obsessive-Compulsive Disorder. Int J Neuropsychopharmcol. 2015;19(2):28. https://dx.doi.org/10.1093/ijnp/pyv084
103. Kasvikis Y, Marks I. Clomipramine, self-exposure, and therapist-accompanied exposure in obsessive-compulsive ritualizers: two-year follow up. J Anxiety Disord [Internet]. 1988; 2:[291‐8 pp.]. Available from: https://www.cochranelibrary.com/central/doi/10.1002/central/CN-00212127/full.
104. Kasvikis Y, Marks IM. Clomipramine in obsessive-compulsive ritualisers treated with exposure therapy: relations between dose, plasma levels, outcome and side effects. Psychopharmacology (Berl). 1988;95(1):113-8. https://dx.doi.org/10.1007/BF00212778
105. Katz R, Landau PDGJHH. Pharmacological responsiveness of dermatitis secondary to compulsive washing letter. Psychiatry-res. 1990;34(2):223‐6.
106. Katz RJ, DeVeaugh-Geiss J. The antiobsessional effects of clomipramine do not require concomitant affective disorder. Psychiatry Res. 1990;31(2):121-9. https://dx.doi.org/10.1016/0165-1781(90)90115-l
107. Kessing LV, Ziersen SC, Andersen FM, Gerds T, Budtz-Jorgensen E. Comparative responses to 17 different antidepressants in major depressive disorder: Results from a 2-year long-term nation-wide population-based study emulating a randomized trial. Acta Psychiatr Scand. 2024;149(5):378-88. https://dx.doi.org/10.1111/acps.13673
108. Khan MNS. Comparison of Escitalopram a new SSRI with TCA, clomipramine in major depressive disorder: A double blind study. Pakistan Journal of Medical Sciences. 2004;20:238-41.
109. Khanna S, Rajendra PN, Channabasavanna SM. Clomipramine in resistant obsessive compulsive disorder. Indian J Psychiatry. 1988;30(4):375-9.
110. Klicpera C, Albert W, Strian F. Effects of somatic treatments on mood in endogenous depression. Acta Psychiatr Scand. 1979;60(2):129-36. https://dx.doi.org/10.1111/j.1600-0447.1979.tb03579.x
111. Klok CJ, Brouwer GJ, van Praag HM, Doogan D. Fluvoxamine and clomipramine in depressed patients. A double-blind clinical study. Acta Psychiatr Scand. 1981;64(1):1-11. https://dx.doi.org/10.1111/j.1600-0447.1981.tb00756.x
112. Koran LM, Pallanti S, Paiva RS, Quercioli L. Pulse loading versus gradual dosing of intravenous clomipramine in obsessive-compulsive disorder. Eur Neuropsychopharmacol. 1998;8(2):121-6. https://dx.doi.org/10.1016/s0924-977x(97)00048-5
113. Kornhaber A, Horwitz IM. A comparison of clomipramine and doxepin in neurotic depression. J Clin Psychiatry. 1984;45(8):337-41.
114. Koszewska I, Rybakowski JK. Antidepressant-induced mood conversions in bipolar disorder: a retrospective study of tricyclic versus non-tricyclic antidepressant drugs. Neuropsychobiology. 2009;59(1):12-6. https://dx.doi.org/10.1159/000202824
115. Kundermann B, Strate P, Hemmeter-Spernal J, Huber MT, Krieg JC, Lautenbacher S. Mid-term effects of serial sleep deprivation therapy implemented in cognitive-behavioral treatment on the neuroendocrine response to clomipramine in patients with major depression. J Psychiatr Res. 2009;43(7):711-20. https://dx.doi.org/10.1016/j.jpsychires.2008.09.004
116. Kupfer DJ, Pollock BG, Perel JM, Miewald JM, Grochocinski VJ, Ehlers CL. Effect of pulse loading with clomipramine on EEG sleep. Psychiatry Res. 1994;54(2):161-75. https://dx.doi.org/10.1016/0165-1781(94)90004-3
117. Kuss HJ, Jungkunz G. Nonlinear pharmacokinetics of chlorimipramine after infusion and oral administration in patients. Progress in Neuro-Psychopharmacology and Biological Psychiatry. 1986;10(6):739-48. https://dx.doi.org/10.1016/0278-5846%2886%2990059-X
118. Landeros-Weisenberger A, Bloch MH, Kelmendi B, Wegner R, Nudel J, Dombrowski P, et al. Dimensional predictors of response to SRI pharmacotherapy in obsessive-compulsive disorder. J Affect Disord. 2010;121(1):175-9. https://dx.doi.org/10.1016/j.jad.2009.06.010
119. Langer G, Aschauer H, Koinig G, Resch F, Schonbeck G. The TSH-response to TRH: A possible predictor of outcome to antidepressant and neuroleptic treatment. Prog Neuropsychopharmacol Biol Psychiatry. 1983;7(2):335-42. https://dx.doi.org/10.1016/0278-5846(83)90121-5
120. Langer G, Koinig G, Hatzinger R, Schonbeck G, Resch F, Aschauer H, et al. Response of thyrotropin to thyrotropin-releasing hormone as predictor of treatment outcome. Prediction of recovery and relapse in treatment with antidepressants and neuroleptics. Arch Gen Psychiatry. 1986;43(9):861-8. https://dx.doi.org/10.1001/archpsyc.1986.01800090047007
121. Langer G, Resch F, Aschauer H, Keshavan MS, Koinig G, Schonbeck G, et al. TSH-response patterns to TRH stimulation may indicate therapeutic mechanisms of antidepressant and neuroleptic drugs. Neuropsychobiology. 1984;11(4):213-8. https://dx.doi.org/10.1159/000118081
122. Larisch R, Klimke A, Hamacher K, Henning U, Estalji S, Hohlfeld T, et al. Influence of synaptic serotonin level on 18F altanserin binding to 5HT2 receptors in man. Behav Brain Res. 2003;139(1):21-9. https://dx.doi.org/10.1016/s0166-4328(01)00412-0
123. Larsen T, Verder H, Christensen JK. Neonatal seizures in maternal clomipramine treatment. Cyclic antidepressives and pregnancy. Ugeskr Laeger. 1984;146(27):2020-1.
124. Lax T, Basoglu M, Marks IM. Expectancy and compliance as predictors of outcome in obsessive-compulsive disorder. Behavioural Psychotherapy. 1992;20:257-66.
125. Lechin F, Van der Dijs B, Gomez F, Arocha L, Acosta E, Lechin E. Distal colon motility as a predictor of antidepressant response to fenfluramine, imipramine and clomipramine. J Affect Disord. 1983;5(1):27-35. https://dx.doi.org/10.1016/0165-0327(83)90033-2
126. Lejoyeux M, Rouillon F, Ades J. Prospective evaluation of the serotonin syndrome in depressed inpatients treated with clomipramine. Acta Psychiatr Scand. 1993;88(5):369-71. https://dx.doi.org/10.1111/j.1600-0447.1993.tb03475.x
127. Leonard H, Swedo S, Rapoport JL, Coffey M, Cheslow D. Treatment of childhood obsessive compulsive disorder with clomipramine and desmethylimipramine: a double-blind crossover comparison. Psychopharmacol Bull. 1988;24(1):93-5.
128. Leonard HL, Meyer MC, Swedo SE, Richter D, Hamburger SD, Allen AJ, et al. Electrocardiographic changes during desipramine and clomipramine treatment in children and adolescents. J Am Acad Child Adolesc Psychiatry. 1995;34(11):1460-8. https://dx.doi.org/10.1097/00004583-199511000-00012
129. Leth-Moller KB, Hansen AH, Torstensson M, Andersen SE, Odum L, Gislasson G, et al. Antidepressants and the risk of hyponatremia: a Danish register-based population study. BMJ Open. 2016;6(5):e011200. https://dx.doi.org/10.1136/bmjopen-2016-011200
130. Licht RW. Is it possible to evaluate true prophylactic efficacy of antidepressants in severely ill patients with recurrent depression? Lessons from a placebo-controlled trial. The fifth trial of the Danish University Antidepressant Group (DUAG-5). J Affect Disord. 2013;148(2):286-90. https://dx.doi.org/10.1016/j.jad.2012.12.009
131. Limosin F, Samuelian JC, Rouillon F. Multicenter double-blind study of the efficacy of paroxetine versus clomipramine in elderly patients with major depression. Journal of Aging and Pharmacotherapy. 2006;13:7-19. https://dx.doi.org/10.1300/J397v13n02_02
132. Linder J, Fyro B, Pettersson U, Werner S. Acute antidepressant effect of lithium is associated with fluctuation of calcium and magnesium in plasma. A double-blind study on the antidepressant effect of lithium and clomipramine. Acta Psychiatr Scand. 1989;80(1):27-36. https://dx.doi.org/10.1111/j.1600-0447.1989.tb01296.x
133. Lykouras L, Markianos M, Hatzimanolis Y. Prolactin and cortisol responses to acute intravenous clomipramine challenge in patients with mania, depression and healthy controls: evidence for reduced serotonergic responsivity. Neuropsychobiology. 2011;63(2):77-81. https://dx.doi.org/10.1159/000323447
134. Ma J, Wang C, Li H, Zhang X, Zhang Y, Hou Y, et al. Cognitive-coping therapy for obsessive-compulsive disorder: a randomized controlled trial. J Psychiatr Res. 2013;47(11):1785‐90. https://dx.doi.org/10.1016/j.jpsychires.2013.08.002
135. Madalena JC. The treatment of depressive states with chlorimipramine by slow intravenous perfusion and oral administration. O Hospital. 1968;74(1):147-56.
136. Maina G, Albert U, Salvi V, Bogetto F. Weight gain during long-term treatment of obsessive-compulsive disorder: a prospective comparison between serotonin reuptake inhibitors. J Clin Psychiatry. 2004;65(10):1365-71. https://dx.doi.org/10.4088/jcp.v65n1011
137. Mao L, Hu M, Luo L, Wu Y, Lu Z, Zou J. The effectiveness of exposure and response prevention combined with pharmacotherapy for obsessive-compulsive disorder: A systematic review and meta-analysis. Front Psychiatr. 2022;13:973838. https://dx.doi.org/10.3389/fpsyt.2022.973838
138. Marazziti D, Pfanner C, Palego L, Gemignani A, Milanfranchi A, Ravagli S, et al. Changes in platelet markers of obsessive-compulsive patients during a double-blind trial of fluvoxamine versus clomipramine. Pharmacopsychiatry. 1997;30(6):245-9. https://dx.doi.org/10.1055/s-2007-979501
139. March JS, Johnston H, Jefferson JW, Kobak KA, Greist JH. Do subtle neurological impairments predict treatment resistance to clomipramine in children and adolescents with obsessive-compulsive disorder? J Child Adolesc Psychopharmacol. 1990;1(2):133-40. https://dx.doi.org/10.1089/cap.1990.1.133
140. Margat P, Broussot T. Testing of G 34-586 in perfusion among 43 patients. Annales Médico-Psychologiques. 1969;1(3):461-8.
141. Marks IM, Lelliott P, Basoglu M, Noshirvani H, Monteiro W, Cohen D, et al. Clomipramine, self-exposure and therapist-aided exposure for obsessive-compulsive rituals. Br J Psychiatry. 1988;152:522-34. https://dx.doi.org/10.1192/bjp.152.4.522
142. Marshall WK, Micev V. The role of intravenous clomipramine in the treatment of obsessional and phobic disorders. Scott Med J. 1975;20(1):49-53. https://dx.doi.org/10.1177/00369330750200S111
143. Mathew SJ, Coplan JD, Perko KA, Goetz RR, de la Neuz M, Hollander E, et al. Neuroendocrine predictors of response to intravenous clomipramine therapy for refractory obsessive-compulsive disorder. Depress Anxiety. 2001;14(4):199-208. https://dx.doi.org/10.1002/da.1067
144. Mavissakalian M, Hamann MS, Jones B. DSM-III personality disorders in obsessive-compulsive disorder: changes with treatment. Compr Psychiatry. 1990;31(5):432-7. https://dx.doi.org/10.1016/0010-440x(90)90028-q
145. Mavissakalian M, Turner SM, Michelson L, Jacob R. Tricyclic antidepressants in obsessive-compulsive disorder: antiobsessional or antidepressant agents? II. Am J Psychiatry. 1985;142(5):572-6. https://dx.doi.org/10.1176/ajp.142.5.572
146. Mawson D, Marks IM, Ramm L. Clomipramine and exposure for chronic obsessive-compulsive rituals: III. Two year follow-up and further findings. Br J Psychiatry. 1982;140:11-8. https://dx.doi.org/10.1192/bjp.140.1.11
147. McClure DJ, Low GL, Gent M. Clomipramine HCL--a double-blind study of a new antidepressant drug. Can Psychiatr Assoc J. 1973;18(5):403-8. https://dx.doi.org/10.1177/070674377301800510
148. McGuire JF, Ung D, Selles RR, Rahman O, Lewin AB, Murphy TK, et al. Treating trichotillomania: a meta-analysis of treatment effects and moderators for behavior therapy and serotonin reuptake inhibitors. J Psychiatr Res. 2014;58:76-83. https://dx.doi.org/10.1016/j.jpsychires.2014.07.015
149. Merino MJ, Gonzalez P, Muniz J, Bobes J. Sexual dysfunction in depressed patients undergoing treatment with antidepressants. Int. 2000;4(4):311-7. https://dx.doi.org/10.1080/13651500050517885
150. Miccoli L, Porro V, Bertolino A. Comparison between the antidepressant activity of S-adenosylmethionine (SAMe) and that of some tricyclic drugs. Acta Neurol (Napoli). 1978;33:243-55.
151. Milanfranchi A, Ravagli S, Lensi P, Marazziti D, Cassano GB. A double-blind study of fluvoxamine and clomipramine in the treatment of obsessive-compulsive disorder. Int Clin Psychopharmacol. 1997;12(3):131-6. https://dx.doi.org/10.1097/00004850-199705000-00002
152. Miller LG, Kraft IA. Psychopharmacologic drug use by 222 patients in the outpatient setting: Side effects in the context of MMPI data. Journal of Pharmacoepidemiology. 1995;4:41-58. https://dx.doi.org/10.1300/J055V04N01_05
153. Minelli A, Bortolomasi M, Scassellati C, Salvoro B, Avesani M, Manganotti P. Effects of intravenous antidepressant drugs on the excitability of human motor cortex: a study with paired magnetic stimulation on depressed patients. Brain Stimul. 2010;3(1):15-21. https://dx.doi.org/10.1016/j.brs.2009.04.003
154. Monteiro WO, Noshirvani HF, Marks IM, Lelliott PT. Anorgasmia from clomipramine in obsessive-compulsive disorder. A controlled trial. Br J Psychiatry. 1987;151:107-12. https://dx.doi.org/10.1192/bjp.151.1.107
155. Montejo AL, Llorca G, Izquierdo JA, Rico-Villademoros F. Incidence of sexual dysfunction associated with antidepressant agents: a prospective multicenter study of 1022 outpatients.Spanish Working Group for the Study of Psychotropic-Related Sexual Dysfunction. J Clin Psychiatry. 2001;62:10-21.
156. Montgomery SA. A meta-analysis of the efficacy and tolerability of paroxetine versus tricyclic antidepressants in the treatment of major depression. Int Clin Psychopharmacol. 2001;16(3):169-78. https://dx.doi.org/10.1097/00004850-200105000-00006
157. Moukaddam NJ, Hirschfeld RM. Intravenous antidepressants: a review. Depress Anxiety. 2004;19(1):1-9. https://dx.doi.org/10.1002/da.10135
158. Moyes I, Ray R, Moyes R. Plasma levels and clinical improvement-a comparative study of clomipramine and amitriptyline in depression. Postgrad Med J. 1980;56:127‐9.
159. Mumoli N, Cocciolo M, Vitale J, Mantellassi M, Sabatini S, Gambaccini L, et al. Diabetes mellitus associated with clomipramine treatment: a retrospective analysis. Acta Diabetol. 2014;51(1):167-8. https://dx.doi.org/10.1007/s00592-013-0500-z
160. Mundo E, Bareggi SR, Pirola R, Bellodi L. Effect of acute intravenous clomipramine and antiobsessional response to proserotonergic drugs: is gender a predictive variable? Biol Psychiatry. 1999;45(3):290-4. https://dx.doi.org/10.1016/s0006-3223(98)00027-4
161. Mundo E, Bareggi SR, Pirola R, Bellodi L, Smeraldi E. Long-term pharmacotherapy of obsessive-compulsive disorder: a double-blind controlled study. J Clin Psychopharmacol. 1997;17(1):4-10. https://dx.doi.org/10.1097/00004714-199702000-00002
162. Mundo E, Bellodi L, Smeraldi E. Effects of acute intravenous clomipramine on obsessive-compulsive symptoms and response to chronic treatment. Biol Psychiatry. 1995;38(8):525-31. https://dx.doi.org/10.1016/0006-3223(94)00373-B
163. Mundo E, Maina G, Uslenghi C. Multicentre, double-blind, comparison of fluvoxamine and clomipramine in the treatment of obsessive-compulsive disorder. Int Clin Psychopharmacol. 2000;15(2):69-76. https://dx.doi.org/10.1097/00004850-200015020-00002
164. Murphy JE. Comparative studies with clomipramine (Anafranil) and clomipramine combinations. J Int Med Res. 1975;3:63-71.
165. Murphy JE. A comparative trial of Anafranil, Pertofran and an Anafranil/Pertofran combination. J Int Med Res. 1977;5(1):16-23.
166. Müller-Oerlinghausen B, Fahndrich E. The relationship between pharmacokinetic data and the clinical response in patients treated with maprotiline or clomipramine by intravenous infusion. Pharmacopsychiatry. 1985;18(1):100-1. http://dx.doi.org/10.1055/s-2007-1017328
167. Möller HJ, Kissling W, Bottermann P. Serial application of clonidine tests during antidepressive treatment with chlorimipramine. Pharmacopsychiatry. 1984;17(6):184-7. https://dx.doi.org/10.1055/s-2007-1017434
168. Möller SE, Bech P, Bjerrum H, Bojholm S, Butler B, Folker H, et al. Plasma ratio tryptophan/neutral amino acids in relation to clinical response to paroxetine and clomipramine in patients with major depression. J Affect Disord. 1990;18(1):59-66. https://dx.doi.org/10.1016/0165-0327(90)90117-q
169. Nagayama H, Nagano K, Ikezaki A, Tashiro T. Prediction of efficacy of antidepressant by 1-week test therapy in depression. J Affect Disord. 1991;23(4):213-6. https://dx.doi.org/10.1016/0165-0327(91)90102-x
170. Nahunek K, Svestka J, Ceskova E. Comparison of onset of the therapeutic effect of desipramine and clomipramine at oral and intravenous application in endogenous depression. Act Nerv Super (Praha). 1984;26(1):29-30.
171. Nielsen NP, Cesana B, Zizolfi S, Ascalone V, Priore P, Morselli PL. Therapeutic effects of fengabine, a new GABAergic agent, in depressed outpatients: a double-blind study versus clomipramine. Acta Psychiatr Scand. 1990;82(5):366-71. https://dx.doi.org/10.1111/j.1600-0447.1990.tb01402.x
172. Ninan PT, Rothbaum BO, Marsteller FA, Knight BT, Eccard MB. A placebo-controlled trial of cognitive-behavioral therapy and clomipramine in trichotillomania. J Clin Psychiatry. 2000;61(1):47-50. https://dx.doi.org/10.4088/jcp.v61n0111
173. Noguera R, Altuna R, Alvarez E, Ayuso JL, Casais L, Udina C. Fluoxetine vs. clomipramine in depressed patients: a controlled multicentre trial. J Affect Disord. 1991;22(3):119-24. https://dx.doi.org/10.1016/0165-0327(91)90045-t
174. O'Flanagan PM, Psych FRC, Bhansali RT. A statistical analysis of 300 cases of affective disorders treated by clomipramine infusion therapy. Journal de Pharmacologie. 1974;5:72.
175. O'Sullivan G, Noshirvani H, Marks I, Monteiro W, Lelliott P. Six-year follow-up after exposure and clomipramine therapy for obsessive compulsive disorder. J Clin Psychiatry. 1991;52(4):150-5.
176. Okayasu H, Ozeki Y, Fujii K, Takano Y, Saeki Y, Hori H, et al. Pharmacotherapeutic determinants for QTc interval prolongation in Japanese patients with mood disorder. Pharmacopsychiatry. 2012;45(7):279-83. https://dx.doi.org/10.1055/s-0032-1308969
177. Okayasu H, Ozeki Y, Fujii K, Takano Y, Shinozaki T, Ohrui M, et al. Investigation of the Proarrhythmic Effects of Antidepressants according to QT Interval, QT Dispersion and T Wave Peak-to-End Interval in the Clinical Setting. Psychiatry Investig. 2019;16(2):159-66. https://dx.doi.org/10.30773/pi.2018.12.11
178. Orgeta V, Tabet N, Nilforooshan R, Howard R. Efficacy of Antidepressants for Depression in Alzheimer's Disease: Systematic Review and Meta-Analysis. J Alzheimers Dis. 2017;58(3):725-33. https://dx.doi.org/10.3233/JAD-161247
179. Pahus K, Henckel J. A comparative study of the effect of Anafranil on cases of depression of a neurotic and endogenous nature. Nord Psykiatr Tidsskr. 1970;24(3):235-8. https://dx.doi.org/10.3109/08039487009101259
180. Pallanti S, Quercioli L, Paiva RS, Koran LM. Citalopram for treatment-resistant obsessive-compulsive disorder. Eur Psychiatry. 1999;14(2):101-6. https://dx.doi.org/10.1016/s0924-9338(99)80725-1
181. Pandey A, Pandey AK, Pandey M, Srivastava M. Clinical correlates of rapid eyeball movement sleep behaviour disorder with clomipramine: A case control study. Journal of Clinical and Diagnostic Research. 2020;14:VC01-VC4. https://dx.doi.org/10.7860/jcdr/2020/45896.14207
182. Pato MT, Pigott TA, Hill JL, Grover GN, Bernstein S, Murphy DL. Controlled comparison of buspirone and clomipramine in obsessive-compulsive disorder. Am J Psychiatry. 1991;148(1):127-9. https://dx.doi.org/10.1176/ajp.148.1.127
183. Pato MT, Zohar-Kadouch R, Zohar J, Murphy DL. Return of symptoms after discontinuation of clomipramine in patients with obsessive-compulsive disorder. Am J Psychiatry. 1988;145(12):1521-5. https://dx.doi.org/10.1176/ajp.145.12.1521
184. Perroud N, Bondolfi G, Uher R, Gex-Fabry M, Aubry JM, Bertschy G, et al. Clinical and genetic correlates of suicidal ideation during antidepressant treatment in a depressed outpatient sample. Pharmacogenomics. 2011;12(3):365-77. https://dx.doi.org/10.2217/pgs.10.189
185. Persson ML, Adler M, Hetta J. Pulse intravenous clomipramine as an alternative antidepressant treatment to ECT. A pilot study. European Journal of Psychiatry. 2007;21:263-7. https://dx.doi.org/10.4321/S0213-61632007000400003
186. Perugi G, Toni C, Frare F, Travierso MC, Hantouche E, Akiskal HS. Obsessive-compulsive-bipolar comorbidity: a systematic exploration of clinical features and treatment outcome. J Clin Psychiatry. 2002;63(12):1129-34.
187. Pigott TA, L'Heureux F, Hill JL, Bihari K, Bernstein SE, Murphy DL. A double-blind study of adjuvant buspirone hydrochloride in clomipramine-treated patients with obsessive-compulsive disorder. J Clin Psychopharmacol. 1992;12(1):11-8. https://dx.doi.org/10.1097/00001573-199202000-00003
188. Pigott TA, Pato MT, Bernstein SE, Grover GN, Hill JL, Tolliver TJ, et al. Controlled comparisons of clomipramine and fluoxetine in the treatment of obsessive-compulsive disorder. Behavioral and biological results. Arch Gen Psychiatry. 1990;47(10):926-32. https://dx.doi.org/10.1001/archpsyc.1990.01810220042005
189. Pigott TA, Pato MT, L'Heureux F, Hill JL, Grover GN, Bernstein SE, et al. A controlled comparison of adjuvant lithium carbonate or thyroid hormone in clomipramine-treated patients with obsessive-compulsive disorder. J Clin Psychopharmacol. 1991;11(4):242-8.
190. Pinder RM, Blum A, Stulemeijer SM, Barres M, Molczadzki M, Rigaud A, et al. A double-blind multicentre trial comparing the efficacy and side-effects of mianserin and chlorimipramine in depressed in- and outpatients. Int Pharmacopsychiatry. 1980;15(4):218-27. https://dx.doi.org/10.1159/000468441
191. Pinkava V, Micev V, Marshall WK. Effect of intravenous clomipramine (Anafranil) treatment as reflected in 16 PF personality test. (A pilot study). J Int Med Res. 1974;2:244-8.
192. Pizarro M, Fontenelle LF, Paravidino DC, Yucel M, Miguel EC, de Menezes GB. An updated review of antidepressants with marked serotonergic effects in obsessive-compulsive disorder. Expert Opin Pharmacother. 2014;15(10):1391-401. https://dx.doi.org/10.1517/14656566.2014.914493
193. Pollock BG, Perel JM, Kupfer DJ, Bowler KA, Miewald JM. Early response patterns associated with successful clomipramine treatment. J Clin Psychopharmacol. 1993;13(6):442-7.
194. Pollock BG, Perel JM, Shostak M, Antelman SM, Brandom B, Kupfer DJ. Understanding the response lag to tricyclics. I. Application of pulse-loading regimens with intravenous clomipramine. Psychopharmacol Bull. 1986;22(1):214-9.
195. Porter RJ, Mulder RT, Joyce PR. Baseline prolactin and L-tryptophan availability predict response to antidepressant treatment in major depression. Psychopharmacology (Berl). 2003;165(3):216-21. https://dx.doi.org/10.1007/s00213-002-1282-0
196. Quilty LC, Godfrey KM, Kennedy SH, Bagby RM. Harm avoidance as a mediator of treatment response to antidepressant treatment of patients with major depression. Psychother Psychosom. 2010;79(2):116-22. https://dx.doi.org/10.1159/000276372
197. Rabe-Jablonska J, Szymanska A. Diurnal profile of melatonin secretion in the acute phase of major depression and in remission. Med Sci Monit. 2001;7(5):946-52.
198. Rachman S, Cobb J, Grey S, McDonald B, Mawson D, Sartory G, et al. The behavioural treatment of obsessional-compulsive disorders, with and without clomipramine. Behav Res Ther. 1979;17(5):467-78. https://dx.doi.org/10.1016/0005-7967(79)90063-9
199. Rack PH. Clinical experience in the treatment of obsessional states (2). J Int Med Res. 1977;5:81-90.
200. Rapisarda V, Bongiorno G. Double blind test with mianserin versus chlorimipramine. Adv Biochem Psychopharmacol. 1982;32:141-9.
201. Rapoport J, Elkins R, Mikkelsen E. Clinical controlled trial of chlorimipramine in adolescents with obsessive-compulsive disorder. Psychopharmacol Bull. 1980;16(3):61-3.
202. Ravizza L, Barzega G, Bellino S, Bogetto F, Maina G. Predictors of drug treatment response in obsessive-compulsive disorder. J Clin Psychiatry. 1995;56(8):368-73.
203. Revet A, Montastruc F, Roussin A, Raynaud JP, Lapeyre-Mestre M, Nguyen TTH. Antidepressants and movement disorders: a postmarketing study in the world pharmacovigilance database. BMC Psychiatry. 2020;20(1):308. https://dx.doi.org/10.1186/s12888-020-02711-z
204. Riemann D, Berger M. The effects of total sleep deprivation and subsequent treatment with clomipramine on depressive symptoms and sleep electroencephalography in patients with a major depressive disorder. Acta Psychiatr Scand. 1990;81(1):24-31. https://dx.doi.org/10.1111/j.1600-0447.1990.tb06444.x
205. Ross S, Fallon BA, Petkova E, Feinstein S, Liebowitz MR. Long-term follow-up study of patients with refractory obsessive-compulsive disorder. J Neuropsychiatry Clin Neurosci. 2008;20(4):450-7. https://dx.doi.org/10.1176/appi.neuropsych.20.4.450 https://dx.doi.org/10.1176/jnp.2008.20.4.450
206. Rothbart R, Amos T, Siegfried N, Ipser JC, Fineberg N, Chamberlain SR, et al. Pharmacotherapy for trichotillomania. Cochrane Database Syst Rev. 2013(11):CD007662. https://dx.doi.org/10.1002/14651858.CD007662.pub2
207. Sallee FR, Koran LM, Pallanti S, Carson SW, Sethuraman G. Intravenous clomipramine challenge in obsessive-compulsive disorder: predicting response to oral therapy at eight weeks. Biol Psychiatry. 1998a;44(3):220-7. https://dx.doi.org/10.1016/s0006-3223(97)00373-9
208. Sallee FR, Pollock BG, Perel JM, Ryan ND, Stiller RL. Intravenous pulse loading of clomipramine in adolescents with depression. Psychopharmacol Bull. 1989;25(1):114-8.
209. Sallee FR, Vrindavanam NS, Deas-Nesmith D, Odom AM, Carson SW, Sethuraman G. Parenteral clomipramine challenge in depressed adolescents: mood and neuroendocrine response. Biol Psychiatry. 1998b;44(7):562-7. https://dx.doi.org/10.1016/s0006-3223(97)00447-2
210. Sanchez-Meca J, Rosa-Alcazar AI, Iniesta-Sepulveda M, Rosa-Alcazar A. Differential efficacy of cognitive-behavioral therapy and pharmacological treatments for pediatric obsessive-compulsive disorder: a meta-analysis. J Anxiety Disord. 2014;28(1):31-44. https://dx.doi.org/10.1016/j.janxdis.2013.10.007
211. Scarzella L, Scarzella R, Mailland F, Bergamasco B. Amineptine in the management of the depressive syndromes. Prog Neuropsychopharmacol Biol Psychiatry. 1985;9(4):429-39. https://dx.doi.org/10.1016/0278-5846(85)90197-6
212. Schlienger RG, Fischer LM, Jick H, Meier CR. Current use of selective serotonin reuptake inhibitors and risk of acute myocardial infarction. Drug Saf. 2004;27(14):1157-65. https://dx.doi.org/10.2165/00002018-200427140-00006
213. Schoretsanitis G, Spigset O, Stingl JC, Deligiannidis KM, Paulzen M, Westin AA. The impact of pregnancy on the pharmacokinetics of antidepressants: a systematic critical review and meta-analysis. Expert Opin Drug Metab Toxicol. 2020;16(5):431-40. https://dx.doi.org/10.1080/17425255.2020.1750598
214. Sepulveda-Lizcano L, Arenas-Villamizar VV, Jaimes-Duarte EB, Garcia-Pacheco H, Paredes CS, Bermudez V, et al. Metabolic Adverse Effects of Psychotropic Drug Therapy: A Systematic Review. European Journal of Investigation in Health Psychology & Education. 2023;13(8):1505-20. https://dx.doi.org/10.3390/ejihpe13080110
215. Serna MC, Cruz I, Real J, Gasco E, Galvan L. Duration and adherence of antidepressant treatment (2003 to 2007) based on prescription database. Eur Psychiatry. 2010;25(4):206-13. https://dx.doi.org/10.1016/j.eurpsy.2009.07.012
216. Shaw DM, Macsweeney DA, Hewland R, Johnson AL. Tricyclic antidepressants and tryptophan in unipolar depression. Psychol Med. 1975;5(3):276-8. https://dx.doi.org/10.1017/s0033291700056646
217. Silva FR, Wijewickrama HS. Clomipramine in phobic and obsessional states: preliminary report. N Z Med J. 1976;84(567):4-6.
218. Singer L. Clinical study of the antidepressive action of chlorimipramine (anafranil). Annales Médico-Psychologiques. 1968;2(2):286-.
219. Skapinakis P, Caldwell D, Hollingworth W, Bryden P, Fineberg N, Salkovskis P, et al. A systematic review of the clinical effectiveness and cost-effectiveness of pharmacological and psychological interventions for the management of obsessive-compulsive disorder in children/adolescents and adults. Health Technol Assess. 2016a;20(43):1-392. https://dx.doi.org/10.3310/hta20430
220. Skapinakis P, Caldwell DM, Hollingworth W, Bryden P, Fineberg NA, Salkovskis P, et al. Pharmacological and psychotherapeutic interventions for management of obsessive-compulsive disorder in adults: a systematic review and network meta-analysis. Lancet Psychiatry. 2016b;3(8):730-9. https://dx.doi.org/10.1016/S2215-0366(16)30069-4
221. Skapinakis P, Caldwell DM, Hollingworth W, Bryden P, Fineberg NA, Salkovskis P, et al. Pharmacological and psychotherapeutic interventions for management of obsessive-compulsive disorder in adults: A systematic review and network meta-analysis. Focus (United States). 2021;19:457-67. https://dx.doi.org/10.1176/APPI.FOCUS.19402
222. Slikboer R, Nedeljkovic M, Bowe SJ, Moulding R. A systematic review and meta‐analysis of behaviourally based psychological interventions and pharmacological interventions for trichotillomania. Clinical Psychologist. 2017;21(1):20-32. 10.1111/cp.12074
223. Soomro GM. Obsessive compulsive disorder. Clin Evid (Online). 2012;18:18.
224. Souetre E, Lozet H, Cimarosti I. Predicting factors for absenteeism in patients with major depressive disorders. Eur J Epidemiol. 1997;13(1):87-93. https://dx.doi.org/10.1023/a:1007397913193
225. Souetre E, Martin P, Lozet H, Monteban H. Quality of life in depressed patients: comparison of fluoxetine and major tricyclic antidepressants. Int Clin Psychopharmacol. 1996;11(1):45-52. https://dx.doi.org/10.1097/00004850-199603000-00006
226. Statens beredning för medicinsk och social utvärdering. Behandling av depressionssjukdomar, volym 1-3. En systematisk litteraturöversikt. SBU-rapport. 2004;166(1-3) Available from: https://www.sbu.se/sv/publikationer/SBU-utvarderar/behandling-av-depressionssjukdomar/
227. Steinert T, Froscher W. Epileptic Seizures Under Antidepressive Drug Treatment: Systematic Review. Pharmacopsychiatry. 2018;51(4):121-35. https://dx.doi.org/10.1055/s-0043-117962
228. Stern RS, Marks IM, Wright J, Luscombe DK. Clomipramine: plasma levels, side effects and outcome in obsessive-compulsive neurosis. Postgrad Med J. 1980;56 Suppl 1:134-9.
229. Suchting R, Tirumalajaru V, Gareeb R, Bockmann T, de Dios C, Aickareth J, et al. Revisiting monoamine oxidase inhibitors for the treatment of depressive disorders: A systematic review and network meta-analysis. J Affect Disord. 2021;282:1153-60. https://dx.doi.org/10.1016/j.jad.2021.01.021
230. Swedo SE, Leonard HL, Rapoport JL, Lenane MC, Goldberger EL, Cheslow DL. A double-blind comparison of clomipramine and desipramine in the treatment of trichotillomania (hair pulling). N Engl J Med. 1989;321(8):497-501. https://dx.doi.org/10.1056/NEJM198908243210803
231. Szegedi A, Wetzel H, Leal M, Hartter S, Hiemke C. Combination treatment with clomipramine and fluvoxamine: drug monitoring, safety, and tolerability data. J Clin Psychiatry. 1996;57(6):257-64.
232. Szymanska A, Rabe-Jablonska J, Karasek M. Diurnal profile of melatonin concentrations in patients with major depression: relationship to the clinical manifestation and antidepressant treatment. Neuroendocrinol Lett. 2001;22(3):192-8.
233. Tao Y, Li H, Li L, Zhang H, Xu H, Zhang H, et al. Comparing the efficacy of pharmacological and psychological treatment, alone and in combination, in children and adolescents with obsessive-compulsive disorder: A network meta-analysis. J Psychiatr Res. 2022;148:95-102. https://dx.doi.org/10.1016/j.jpsychires.2022.01.057
234. Taylor D, Poulou S, Clark I. The cardiovascular safety of tricyclic antidepressants in overdose and in clinical use. Therapeutic Advances in Psychopharmacology. 2024;14: https://dx.doi.org/10.1177/20451253241243297
235. Thoren P, Asberg M, Cronholm B, Jornestedt L, Traskman L. Clomipramine treatment of obsessive-compulsive disorder. I. A controlled clinical trial. Arch Gen Psychiatry. 1980;37(11):1281-5. https://dx.doi.org/10.1001/archpsyc.1980.01780240079009
236. Uguz F. The Use of Antidepressant Medications During Pregnancy and the Risk of Neonatal Seizures: A Systematic Review. J Clin Psychopharmacol. 2019;39(5):479-84. https://dx.doi.org/10.1097/JCP.0000000000001093
237. Uguz F. The Relationship Between Maternal Antidepressants and Neonatal Hypoglycemia: A Systematic Review. Alpha Psychiatry. 2021;22(5):224-9. https://dx.doi.org/10.1530/alphapsychiatry.2021.21143
238. Ulrich G, Haug HJ, Fahndrich E. Acute vs. chronic EEG effects in maprotiline- and in clomipramine-treated depressive inpatients and the prediction of therapeutic outcome. J Affect Disord. 1994;32(3):213-7. https://dx.doi.org/10.1016/0165-0327(94)90020-5
239. Ulrich G, Haug HJ, Stieglitz RD, Fahndrich E. EEG characteristics of clinically defined on-drug-responders and non-responders--a comparison clomipramine vs. maprotiline. Pharmacopsychiatry. 1988;21(6):367-8. https://dx.doi.org/10.1055/s-2007-1017011
240. van Kammen DP, van Scheyen JD, Murphy DL. Platelet monoamine oxidase activity and clomipramine-induced mania in unipolar depressed patients. Biol Psychiatry. 1980;15(4):565-73.
241. van Scheyen JD, van Kammen DP. Clomipramine-induced mania in unipolar depression. Arch Gen Psychiatry. 1979;36(5):560-5. https://dx.doi.org/10.1001/archpsyc.1979.01780050070008
242. Van Scheyen JD, Van Praag HM, Korf J. Controlled study comparing nomifensine and clomipramine in unipolar depression, using the probenecid technique. Br J Clin Pharmacol. 1977;4:179S-84S. https://dx.doi.org/10.1111/j.1365-2125.1977.tb05749.x
243. van Soest EM, Dieleman JP, Siersema PD, Schoof L, Sturkenboom MC, Kuipers EJ. Tricyclic antidepressants and the risk of reflux esophagitis. Am J Gastroenterol. 2007;102(9):1870-7. https://dx.doi.org/10.1111/j.1572-0241.2007.01320.x
244. Varigonda AL, Jakubovski E, Bloch MH. Systematic Review and Meta-Analysis: Early Treatment Responses of Selective Serotonin Reuptake Inhibitors and Clomipramine in Pediatric Obsessive-Compulsive Disorder. J Am Acad Child Adolesc Psychiatry. 2016;55(10):851-9.e2. https://dx.doi.org/10.1016/j.jaac.2016.07.768
245. Veale D, Miles S, Smallcombe N, Ghezai H, Goldacre B, Hodsoll J. Atypical antipsychotic augmentation in SSRI treatment refractory obsessive-compulsive disorder: a systematic review and meta-analysis. BMC Psychiatry. 2014;14:317. https://dx.doi.org/10.1186/s12888-014-0317-5
246. Vencovsky E. Therapeutic experience with chlorimipramine injections. Act Nerv Super (Praha). 1971;13(3):161-2.
247. Vestergaard P, Rejnmark L, Mosekilde L. Selective serotonin reuptake inhibitors and other antidepressants and risk of fracture. Calcif Tissue Int. 2008;82(2):92-101. https://dx.doi.org/10.1007/s00223-007-9099-9
248. Viktorin A, Uher R, Reichenberg A, Levine SZ, Sandin S. Autism risk following antidepressant medication during pregnancy. Psychol Med. 2017;47(16):2787-96. https://dx.doi.org/10.1017/S0033291717001301
249. Voican CS, Martin S, Verstuyft C, Corruble E, Perlemuter G, Colle R. Liver function test abnormalities in depressed patients treated with antidepressants: A real-world systematic observational study in psychiatric settings. PLoS ONE. 2016;11 https://dx.doi.org/10.1371/journal.pone.0155234
250. Volavka J, Neziroglu F, Yaryura-Tobias JA. Clomipramine and imipramine in obsessive-compulsive disorder. Psychiatry Res. 1985;14(1):85-93. https://dx.doi.org/10.1016/0165-1781(85)90092-7
251. Volmat R, Allers G, Vittouris N. Clomipramine or anafranil: Treatment of 100 depressive states. L'Encéphale: Revue de psychiatrie clinique biologique et thérapeutique. 1968;57(2):116-42.
252. Von Oefele K, Grohmann R, Ruther E. Adverse drug reactions in combined tricyclic and MAOI therapy. Pharmacopsychiatry. 1986;19(4):243-4. https://dx.doi.org/10.1055/s-2007-1017216
253. Vos CF, Ter Hark SE, Schellekens AFA, Spijker J, van der Meij A, Grotenhuis AJ, et al. Effectiveness of Genotype-Specific Tricyclic Antidepressant Dosing in Patients With Major Depressive Disorder: A Randomized Clinical Trial. JAMA netw. 2023;6(5):e2312443. https://dx.doi.org/10.1001/jamanetworkopen.2023.12443
254. Warneke LB. Intravenous clomipramine for OCD. Can J Psychiatry. 1992;37(7):522-3.
255. Waxman D. A clinical trial of clomipramine and diazepam in the treatment of phobic and obsessional illness. J Int Med Res. 1977;5:99-110.
256. Wilson K, Mottram P. A comparison of side effects of selective serotonin reuptake inhibitors and tricyclic antidepressants in older depressed patients: a meta-analysis. Int J Geriatr Psychiatry. 2004;19(8):754-62. https://dx.doi.org/10.1002/gps.1156
257. Winkler D, Grohmann R, Friedrich ME, Toto S, Bleich S, Seifert J, et al. Urological adverse drug reactions of psychotropic medication in psychiatric inpatients - A drug surveillance report from German-speaking countries. J Psychiatr Res. 2021;144:412-20. https://dx.doi.org/10.1016/j.jpsychires.2021.10.026
258. Wyndowe J, Solyom L, Ananth J. Anafranil in obsessive compulsive neurosis. Curr Ther Res Clin Exp. 1975;18(5):611-7.
259. Wålinder J, Carlsson A, Persson R. 5-HT reuptake inhibitors plus tryptophan in endogenous depression. Acta Psychiatr Scand Suppl. 1981;290:179-90. https://dx.doi.org/10.1111/j.1600-0447.1981.tb00719.x
260. Wålinder J, Skott A, Carlsson A, Nagy A, Bjorn-Erik R. Potentiation of the antidepressant action of clomipramine by tryptophan. Arch Gen Psychiatry. 1976;33(11):1384-9. https://dx.doi.org/10.1001/archpsyc.1976.01770110112012
261. Xiong GL, Jiang W, Clare R, Shaw LK, Smith PK, Mahaffey KW, et al. Prognosis of patients taking selective serotonin reuptake inhibitors before coronary artery bypass grafting. Am J Cardiol. 2006;98(1):42-7. https://dx.doi.org/10.1016/j.amjcard.2006.01.051
262. Xu J, Hao Q, Qian R, Mu X, Dai M, Wu Y, et al. Optimal Dose of Serotonin Reuptake Inhibitors for Obsessive-Compulsive Disorder in Adults: A Systematic Review and Dose-Response Meta-Analysis. Front Psychiatr. 2021;12:717999. https://dx.doi.org/10.3389/fpsyt.2021.717999
263. Yamada K, Yagi G, Kanba S. Clinical efficacy of tandospirone augmentation in patients with major depressive disorder: a randomized controlled trial. Psychiatry Clin Neurosci. 2003;57(2):183-7. https://dx.doi.org/10.1046/j.1440-1819.2003.01099.x
264. Younus S, Havel L, Stiede JT, Rast CE, Saxena K, Goodman WK, et al. Pediatric Treatment-Resistant Obsessive Compulsive Disorder: Treatment Options and Challenges. Paediatr Drugs. 2024;26(4):397-409. https://dx.doi.org/10.1007/s40272-024-00639-5
265. Zahn TP, Insel TR, Murphy DL. Psychophysiological changes during pharmacological treatment of patients with obsessive compulsive disorder. Br J Psychiatry. 1984;145:39-44. https://dx.doi.org/10.1192/bjp.145.1.39
266. Zapletalek M, Zbytovsky J, Kudrnova K. Clinical experience with maprotilin and maprotilin/clomipramine infusions in resistant depression. Act Nerv Super (Praha). 1982;24(2):73-6.
267. Zhao J. A control study of clomipramine and amitriptyline for treating obsessive-compulsive disorder. Chinese journal of neurology and psychiatry. 1991;24(2):68‐70.
268. Zhou S, Li P, Lyu X, Lai X, Liu Z, Zhou J, et al. Efficacy and dose-response relationships of antidepressants in the acute treatment of major depressive disorders: a systematic review and network meta-analysis. Chin Med J. 2024;20:20. https://dx.doi.org/10.1097/CM9.0000000000003138
269. Zohar J, Insel TR, Zohar-Kadouch RC, Hill JL, Murphy DL. Serotonergic responsivity in obsessive-compulsive disorder. Effects of chronic clomipramine treatment. Arch Gen Psychiatry. 1988;45(2):167-72. https://dx.doi.org/10.1001/archpsyc.1988.01800260081011
270. Zohar J, Judge R. Paroxetine versus clomipramine in the treatment of obsessive-compulsive disorder. OCD Paroxetine Study Investigators. Br J Psychiatry. 1996;169(4):468-74. https://dx.doi.org/10.1192/bjp.169.4.468
